# Magnetic Composite Biomaterials for Neural Regeneration

**DOI:** 10.3389/fbioe.2019.00179

**Published:** 2019-07-25

**Authors:** Jessica L. Funnell, Bailey Balouch, Ryan J. Gilbert

**Affiliations:** Department of Biomedical Engineering, Center for Biotechnology and Interdisciplinary Studies, Rensselaer Polytechnic Institute, Troy, NY, United States

**Keywords:** biomaterials, magnetic nanoparticles, neural regeneration, iron oxide nanoparticles, nervous system injury

## Abstract

Nervous system damage caused by physical trauma or degenerative diseases can result in loss of sensory and motor function for patients. Biomaterial interventions have shown promise in animal studies, providing contact guidance for extending neurites or sustained release of various drugs and growth factors; however, these approaches often target only one aspect of the regeneration process. More recent studies investigate hybrid approaches, creating complex materials that can reduce inflammation or provide neuroprotection in addition to stimulating growth and regeneration. Magnetic materials have shown promise in this field, as they can be manipulated non-invasively, are easily functionalized, and can be used to mechanically stimulate cells. By combining different types of biomaterials (hydrogels, nanoparticles, electrospun fibers) and incorporating magnetic elements, magnetic materials can provide multiple physical and chemical cues to promote regeneration. This review, for the first time, will provide an overview of design strategies for promoting regeneration after neural injury with magnetic biomaterials.

## Introduction

Nervous system injury caused by physical trauma to the brain, spinal cord, or peripheral nerves results in loss of neural function. There are ~17,000 new spinal cord injury (SCI) cases each year in the United States, and <1% experience full neurological recovery (NSCISC, 2019). There are an estimated 1.7 million new traumatic brain injury (TBI) cases each year in the United States (Frati et al., 2017; Reis et al., 2017), with 50% experiencing long-term deficits (Frati et al., 2017). While peripheral nerve injuries are more common and carry significant clinical relevance (Alvites et al., 2018), incidence is difficult to estimate; however, ~2–3% of Level I Emergency Department intakes involve peripheral nerve trauma (Robinson, 2000). Successful nerve regeneration and full functional recovery after severe nerve injury is rare, with few FDA-approved therapies available for patients (Arslantunali et al., 2014). Innovative biomaterial-based therapies are rapidly emerging for improving outcomes after nerve injury. Various hydrogels, nanoparticles, and fibrous materials have been developed and explored by researchers to provide physical and chemical cues to cells, promoting regeneration (Ziemba and Gilbert, 2017). In this review, for the first time, we summarize recent advances in the design of magnetic biomaterials for promoting neural regeneration. First, we describe the pathophysiology of traumatic injury in both the central nervous system (CNS) and peripheral nervous system (PNS), as well as the properties of magnetic materials and their importance in biomaterial design. We then highlight studies utilizing different strategies for neural regeneration with magnetic biomaterials, namely drug and gene delivery, cell stimulation and transplantation, and combinations of both. The review ends with a discussion about the challenges that must be addressed before magnetic biomaterials as neural injury therapies can become clinically feasible, and the direction in which future studies in the field are headed.

Key differences exist between the CNS and PNS in their response to injury, most notably the cellular response, changes in the extracellular matrix (ECM), and capacity for functional regeneration (Vargas and Barres, 2007; Huebner and Strittmatter, 2009; Alvites et al., 2018; Quraishe et al., 2018; Zigmond and Echevarria, 2019). Here, we describe the typical events following trauma to the CNS and PNS and their implications for regenerative outcomes.

### Central Nervous System Injury Pathophysiology

CNS tissue is particularly limited in its capacity for regeneration (Huebner and Strittmatter, 2009; Bollaerts et al., 2017). Following SCI or TBI, a complex multicellular cascade is initiated, which propagates damage for weeks to months after the initial trauma. Physical trauma is sustained to the brain or spinal cord, resulting in hemorrhage and necrosis of neurons and glia. The blood-brain barrier (BBB) becomes compromised and an initially neutrophil-mediated inflammatory response is mounted at the injury epicenter (Karve et al., 2016; Reis et al., 2017; Ziemba and Gilbert, 2017; Zuidema et al., 2018; Albayar et al., 2019). Hours to weeks post-trauma, persistent inflammatory events cause continued insult to the tissue surrounding the injury site, known as secondary injury. Recruited macrophages and resident microglia polarize to predominantly an M1, or pro-inflammatory, state, releasing inflammatory cytokines and reactive oxygen species (ROS) as they phagocytose debris in the injury site (Popovich et al., 1999; Karve et al., 2016; Bollaerts et al., 2017; Kong and Gao, 2017; Reis et al., 2017; Ziemba and Gilbert, 2017). Calcium homeostasis is also affected in response to changes in BBB permeability, which, along with ROS and inflammatory cytokines, leads to cell death via apoptosis of neuronal and glial cells (Liu et al., 1997; Oyinbo, 2011; Karve et al., 2016; Frati et al., 2017; Reis et al., 2017; Ziemba and Gilbert, 2017; Quraishe et al., 2018). Axons proximal to the injury site become demyelinated, as oligodendrocytes have a heightened susceptibility to toxic factors (Casha et al., 2001; Almad et al., 2011). Distal axons are lost due to Wallerian axonal degeneration, leading to further oligodendrocyte apoptosis and accumulation of myelin debris (Vargas and Barres, 2007; Burda et al., 2016; Frati et al., 2017).

Driven by the M1 macrophage-microglial response, astrocytes assume a reactive phenotype and promote the formation of an astrogliotic scar around the injury site (Karve et al., 2016; Kong and Gao, 2017; Liddelow and Barres, 2017; Alizadeh et al., 2019; Bellver-Landete et al., 2019). The scar serves to physically seal off the injury lesion and protect healthy tissue; however, it also acts as a physical and chemical barrier which prevents axonal regeneration through the injury site (McKeon et al., 1991; Karve et al., 2016; Zuidema et al., 2018; Huang et al., 2019). Chondroitin sulfate proteoglycans (CSPGs) are deposited in the ECM of the scar, some of which act as potent inhibitors of axon outgrowth and oligodendrocyte regeneration (Canning et al., 1996; Gilbert et al., 2005; Quraishe et al., 2018; Alizadeh et al., 2019). Taken together, these factors contribute to a chemical and physical barrier that hinders neural regeneration at the injury site (Vargas and Barres, 2007; Reis et al., 2017; Quraishe et al., 2018).

### Peripheral Nervous System Injury Pathophysiology

Distinct differences exist in the pathophysiological response to trauma sustained to the PNS compared to the CNS. These differences may explain why functional regeneration is possible in the PNS (Vargas and Barres, 2007; Alvites et al., 2018; Quraishe et al., 2018; Zigmond and Echevarria, 2019). Peripheral nerve trauma first leads to demyelination of axons by Schwann cells (Rotshenker, 2011; Menorca et al., 2013). Unlike oligodendrocytes, which die by apoptosis upon loss of contact with axons (Barres et al., 1993; Vargas and Barres, 2007), Schwann cells shift to a non-myelinating regenerative phenotype, and proceed to produce neurotrophic factors and recruit macrophages to the injury site (Vargas and Barres, 2007; Rotshenker, 2011; Menorca et al., 2013). The blood-nerve barrier (BNB) can become compromised, allowing macrophages into the injury site, where they produce factors supporting Schwann cell proliferation and phagocytosis of debris (Menorca et al., 2013).

Unlike CNS injury, in which proximal axons are progressively demyelinated (Casha et al., 2001; Almad et al., 2011), minimal proximal demyelination or degeneration is observed in the PNS, and proximal neurons instead shift to a regenerative phenotype (Menorca et al., 2013; Lutz et al., 2017; Alvites et al., 2018; Zigmond and Echevarria, 2019). As the distal axon fragment degenerates, Wallerian degeneration quickly occurs in the distal segment, and myelin debris is cleared by macrophage and Schwann cell phagocytosis to create a favorable environment for axonal regeneration (Vargas and Barres, 2007; Rotshenker, 2011; Alvites et al., 2018; Zigmond and Echevarria, 2019). By contrast, oligodendrocytes in the CNS do not participate in the clearance of myelin debris, entering apoptosis as the axon degenerates, so, myelin debris removal is incomplete and occurs slowly in the CNS (Vargas and Barres, 2007). Effective removal of myelin debris is crucial for regeneration, as there are several myelin-derived inhibitors of axon outgrowth (McKerracher et al., 1994; Mukhopadhyay et al., 1994; Fournier et al., 2001; Vargas and Barres, 2007; Zigmond and Echevarria, 2019).

Once debris has been cleared, Schwann cells proliferate, due largely to macrophage-derived cues, and reconstruct the ECM to form pathways for axons to regenerate. Schwann cells further support regeneration by secreting pro-regenerative growth factors and embedding neural cell adhesion molecules in the endoneurial connective tissue. This is important because functional re-innervation is dependent on the growth cone of the regenerating axon reaching the endoneurial tube before meeting its target. Axon outgrowth triggers a phenotypic switch in Schwann cells, promoting a remyelinating phenotype as regeneration occurs (Alvites et al., 2018).

## Magnetic Materials and Their Properties

The natural regeneration process is unable to provide full functional recovery after severe injury, even in the PNS where regeneration is more successful than in the CNS. Biomaterial-based strategies have been used in both the CNS and PNS to provide local and sustained release of therapeutic molecules as well as provide contact guidance for regenerating cells (Nectow et al., 2012; Ziemba and Gilbert, 2017; Cangellaris and Gillette, 2018). Magnetic nanoparticles (MNPs) have recently been studied as potential biomaterial candidates for their wide variety of biomedical applications: drug delivery, cell labeling and targeting, magnetic resonance imaging (MRI) contrast agents, hyperthermia ablation of tumor cells, and magnet-assisted transfection (Gupta and Gupta, 2005). For neural regeneration applications, MNPs have been studied as drug carriers and cell actuators to generate magnetically responsive therapies that can be non-invasively manipulated inside the body by external magnets. This section will describe the properties of MNPs and how they are important for designing biomedical therapies.

Magnetic properties arise from electrons spinning around the nuclei of atoms and electrons spinning on their axes. The degree to which a material is magnetic depends on the atomic structure of the material and the temperature. In certain atoms, like iron, nickel, and cobalt, magnetic dipoles generated by spinning electrons do not cancel each other out, creating a permanent dipole. The strength of the permanent dipole is termed the magnetic moment, which is a measure of the capacity of the dipole to align itself with an external magnetic field (Estelrich et al., 2015; D'Agata et al., 2017).

A group of adjacent atoms that have their dipoles aligned in the same direction is called a domain. A ferromagnetic material has many domains that orient in different directions but will align in the presence of an external magnetic field. The magnetic properties of ferromagnetic materials change as physical size is reduced. When the size of a ferromagnetic material is lower than a critical diameter (usually around 100–200 nm), the material becomes a single domain. By reducing the diameter even further, the material becomes superparamagnetic (Estelrich et al., 2015). Superparamagnetic materials do not retain their magnetic properties when an external magnetic field is removed, while ferromagnetic materials do. This is an important property for biomedical applications as this prevents attraction between particles, limiting particle agglomeration (D'Agata et al., 2017). Due to their nanoscale size, superparamagnetic nanoparticles can reach their saturation magnetization in the presence of an external magnetic field, resulting in a high magnetic moment. However, a particle's magnetic moment is partially dependent on its volume, with larger volume resulting in a larger magnetic attraction. Therefore, larger particles that are still within the superparamagnetic regime are preferred (Estelrich et al., 2015). Nanoparticle size must also be considered in terms of biocompatibility. Larger particles (diameter >200 nm) are filtered by the spleen and eventually broken down by phagocytic cells, while smaller particles (diameter <10 nm) are rapidly removed via extravasation and renal clearance. Particles ranging from 10 to 100 nm in diameter are usually optimal for intravenous injection, since they are small enough to evade the body's reticuloendothelial system but also penetrate small capillaries (Gupta and Gupta, 2005).

The most common magnetic materials used for biomedical applications are the iron oxides magnetite (Fe_3_O_4_) and maghemite (γ-Fe_2_O_3_). The two are very similar in structure, and therefore have similar properties. They differ slightly in their saturation magnetization values (92–100 emu/g for magnetite and 60–80 emu/g for maghemite) and superparamagnetic diameters (25 nm for magnetite and 30 nm for maghemite) (Estelrich et al., 2015). Other magnetic materials, such as cobalt and chromium, are highly toxic and therefore are commonly avoided for use in biomedical applications. Iron-based metal oxides such as CoFe_2_O_4_, NiFe_2_O_4_, and MnFe_2_O_4_ may be used as the core of the nanoparticle to provide increased magnetic susceptibility; however, non-permeable coatings are necessary to prevent the toxic metals from leaching out into surrounding tissue (McBain et al., 2008; Kudr et al., 2017).

Magnetite and maghemite are advantageous materials because they exhibit chemical stability in physiological conditions, low toxicity, and high magnetic susceptibility. “Naked” superparamagnetic iron oxide nanoparticles (SPIONs) are usually hydrophobic due to a thin layer of surfactant used to synthesize stable particles in an aqueous solution, but particles can be modified with tailored surface chemistry to further improve their stability as well as offer functionalization with ligands or drugs for specific biomedical applications (D'Agata et al., 2017). The synthesis and surface functionalization of magnetic nanoparticles has been extensively reviewed (Gupta and Gupta, 2005; McBain et al., 2008; Estelrich et al., 2015; Hola et al., 2015). This review will focus on the application of these functionalization strategies for neural regeneration in both the CNS and PNS.

## Magnetic Drug and Gene Delivery Systems for Promoting Neural Regeneration

Due to the many advantages of magnetic nanoparticles, numerous strategies have been developed to use these particles to deliver drugs or genes to specific cells. There are various approaches that utilize the magnetic properties of SPIONs to assist in drug or gene delivery: liposome or micelle encapsulation, polymer coatings, and direct surface functionalization. The following sections will focus specifically on using these delivery systems for neural regeneration applications.

### Drug Delivery to the Nervous System via Magnetic Nanoparticles

Early studies using SPIONs for drug delivery in the nervous system looked at how different magnetic liposomes and polymer-encapsulated MNPs affect neurons *in vitro*. Kim et al. fabricated magnetic liposomes from oleic acid-coated SPIONs with a polyethylene glycol (PEG) chain attached to a phospholipid tail. PC12 cells, a rat-derived cell line commonly used to study neuronal differentiation and growth, were exposed to different concentrations of the liposomes for up to 5 d. The magnetic liposomes increased neurite outgrowth in a dose-dependent manner when in the presence of exogeneous nerve growth factor (NGF). Phosphorylated ERK1/2 (associated with the NGF signaling pathway), β-tubulin, and integrin β1 were also upregulated when PC12 cells were incubated with the liposomes and NGF (Kim et al., 2011). Another study looked at magnetic microspheres fabricated by encapsulating magnetite particles and NGF in alginate. Characterization of the release from the microspheres showed that almost all the NGF was released after 72 h. Microspheres were internalized by PC12 cells without influencing viability or differentiation. To demonstrate spatial control of the microspheres, a permanent magnet was placed on the side of the well, attracting the microspheres to one location after being added to the media. Morphological analysis of cells at different distances from the magnet showed that an NGF gradient was established, with the extent of neurite outgrowth following the trend of the gradient (Ciofani et al., 2009). Zuidema et al. directed primary rat dorsal root ganglion (DRG) neurite outgrowth by creating a gradient of NGF released from MNPs. SPIONs were coated with poly-L-lactic acid (PLLA) containing 3 ng/mL NGF. Release characterization showed there was an initial burst release of 1.1 ng in the first 24 h, which then leveled off, releasing nanograms of NGF for 7 d. They then combined the NGF-MNPs with aligned PLLA microfibers to induce directed neurite outgrowth preferentially on one side of the DRG, creating a hybrid biomaterial system that alters neurite extension. The NGF gradient was produced by holding a neodymium magnet 5 mm away from the DRG body, attracting the NGF-MNPs. The neurites extended along the fibers in either direction, but were significantly longer on the side closest to the NGF particles (Zuidema et al., 2015).

A major challenge to neural regeneration in both the peripheral and central nervous systems is the local and sustained delivery of bioactive factors. Systemic administration can be toxic and growth factors are not stable for long periods after injection. Conjugating growth factors to SPIONs has shown to increase their half-life while also maintaining function. One group was able to adsorb brain-derived neurotrophic factor (BDNF) to 60 nm-diameter magnetite nanoparticles at 70% efficiency. Using an *in vitro* model of the BBB, they showed that an external magnet was able to induce transportation of 3.5 times more adsorbed BDNF via MNPs compared to free, un-adsorbed BDNF across the *in vitro* BBB model. They also showed that transport of the BDNF-MNPs did not interfere with the integrity of their BBB model (Pilakka-Kanthikeel et al., 2013). Another study looked at the conjugation of NGF to MNPs. Marcus et al. coated maghemite nanoparticles with human serum albumin and covalently attached a PEG linker terminated with N-hydroxysuccinimide, which was used to covalently bind ~70 NGF molecules per particle. Active NGF levels remained constant for the NGF-MNPs over 7 d when cultured with PC12 cells, while free NGF (at same starting concentration as the NGF-MNPs) completely degraded. The NGF-MNPs also increased neurite length, the number of neurite branch points, and expression levels of neuronal differentiation markers in the PC12 cells compared to free NGF at the same concentration (Marcus et al., 2014). Ziv-Polat et al. compared the conjugation of three different neurotrophic factors: NGF, glial-derived neurotrophic factor (GDNF), and fibroblast growth factor-2 (FGF-2). SPIONs were coated with gelatin that was functionalized with activated double bonds to allow covalent binding of one of the three growth factors. The neurotrophic factors conjugated to SPIONs showed a significant increase in stability compared to the free factors alone both in medium and in tissue cultures over the course of 10 d. The stability of GDNF conjugated to SPIONs increased the most out of the three factors compared to its corresponding un-conjugated factor. SPION-conjugated GDNF retained about 90% of its initial concentration as opposed to free GDNF, which only retained 30% of its initial concentration. The GDNF-MNPs also had the greatest effect on the onset of myelination in the DRG cultures; myelin was detected 7 d earlier than in cultures incubated with free GDNF and 21 d earlier than in control cultures. Transmission electron micrographs (TEMs) show that GDNF-MNPs were internalized by both neurons and Schwann cells in the DRG cultures (Ziv-Polat et al., 2014).

When choosing a functionalization strategy, the target tissue as well as the subject of therapeutic intervention should be considered. Using drug-carrying MNPs to target the peripheral nerve will require different modifications than particles designed to pass the BBB or blood-cerebral spinal fluid (CSF) barrier. These considerations will also vary based on the pathophysiology of the target area; MNPs may enter the lesion site after spinal cord injury or traumatic brain injury because of the damaged vasculature at these areas, whereas particles delivering an Alzheimer's drug may need functionalization to allow for receptor-mediated transcytosis through the BBB. The study by Jeffery et al. shows that MNPs delivered intravenously can localize at the lesion site at both 15 min and 6 h after hemi-section SCI without magnetic field guidance, corresponding with blood-CSF barrier damage (Jeffery et al., 2009). Kong et al. showed that 124-nm diameter polystyrene-coated magnetite particles will not cross the BBB alone, but will under the influence of an external magnet placed at the head of the mouse. Most of the particles were cleared from circulation and localized in the liver and spleen, but 30% of the particles were still localized in the brain after 48 h (Kong et al., 2012). The next section will discuss the different strategies for delivering drugs to the brain using MNPs.

#### Magnetic Drug Delivery Systems Targeting the Brain

Strategies to improve passage of pharmaceutical agents across the BBB is an ongoing area of research. MNP-based approaches have received a lot of attention because of their magnetic susceptibility, improving site-specific delivery with the aid of an external magnetic field. Several reviews summarize different strategies to enhance the ability of MNPs to cross the BBB for applications including MRI imaging and tumor hyperthermia (Dilnawaz and Sahoo, 2015; Thomsen et al., 2015; D'Agata et al., 2017). The combination of surface group functionalization with the magnetic susceptibility of MNPs is also a promising approach for drug delivery to the brain. Wen et al. and Aguilera et al. tested the *in vitro* potential of a magnetic drug delivery system to affect endothelial cells, a major component of the BBB. Wen et al. fabricated magnetic poly(lactide-co-glycolide) (PLGA)/lipid liposomes with a quantum dot core coated with PEG and conjugated with trans-activating transcriptor (TAT) peptides. They were able to individually encapsulate the drugs hesperidin, naringin, and glutathione within the liposomes with loading capacities exceeding 10% and encapsulation efficiencies exceeding 90%. The liposomes released 20–40% of each drug within the first 24 h, and about 90% was released over the course of 8 d. The TAT-conjugated liposomes exhibited a slightly prolonged release compared to control liposomes. This study demonstrated that bEnd.3 cells, an endothelial brain cell line, accumulated higher levels of TAT-liposomes than control liposomes; therefore, they claimed that the TAT-conjugated liposomes will be more efficient at delivering drugs to the brain *in vivo* (Wen et al., 2014). Aguilera et al. looked at the effect of dopamine-loaded carboxymethyl cellulose-coated magnetite nanoparticles on human lung microvascular endothelial cell cultures. Nearly 40% of the dopamine was released in 1 h, with the rest releasing more slowly over the course of 4 h. The MNPs crossed the barrier of cells cultured on a transwell without magnetic field stimulation and without altering the barrier integrity (Aguilera et al., 2019). These studies demonstrate that chemical surface modifications can improve MNP passage through the BBB. Magnetic targeting with an external magnet can further improve this efficacy *in vivo*. Examples of this are described below.

**Figure 1:**
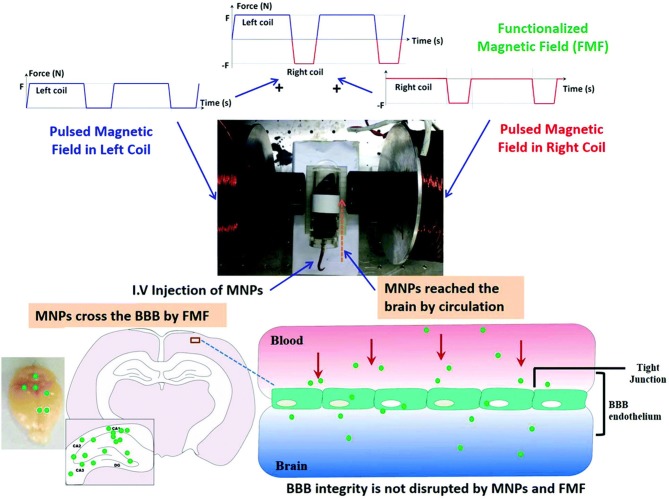
Republished with permission of RSC Pub and the Royal Society of Chemistry from Amin et al. (2017) via Copyright Clearance Center. Schematic representation of the differential effects of the functionalized magnetic field on the transport of magnetic particles across the intact BBB in normal mice. The functionalized magnetic field was generated using an electromagnetic actuator, and a 10-min exposure time was used for each experiment. The magnetic particles successfully crossed the BBB and reached the brain under all observed functionalized magnetic field conditions. No histological changes or neurotoxicity in the brain was observed after the experiments. Moreover, BBB integrity was not disrupted by magnetic particle administration and the functionalized magnetic field.

The ability of SPIONs to enter the brain without disturbing the integrity of the BBB is an important property for drug delivery. The types of drugs that can cross the BBB without modification is limited to small lipophilic molecules, while other brain delivery strategies rely on transient loosening of brain endothelial tight junctions (D'Agata et al., 2017). An external magnetic field generated by a 0.5-T magnet held over the brain for 1 h after intravenous injection allowed magnetic liposomes to penetrate the BBB and be taken up by neural cells; however, the mechanism behind this phenomenon is still unknown. The study also demonstrated that intra-arterial injection of the same liposomes loaded with magnetite nanoparticles and paclitaxel increased the paclitaxel content in the brain by 1.5-fold, even though the intra-arterial dose was 10% of the intravenous dose (Zhao et al., 2012). Another study compared BBB crossing efficiency of MNPs with different magnetic field strengths. Specifically, the effects of a positive- and negative-pulsed magnetic field generated by electromagnetic coils was investigated on delivery efficiency of dextran-coated magnetite SPIONs loaded with osmotin across the BBB, as depicted in Figure 1. A range of magnetic field conditions were tested, namely coil currents of 3 or 6 A, alternating frequencies of 0.5 or 1.0 Hz, and exposure times of 5 or 10 min. Significantly more MNPs reached the brain when 6 A, 0.5 Hz, and a 10-min exposure time was used than any other combination of conditions. The MNPs did not produce any evidence of toxicity in the brain nor did they disrupt the brain-endothelial barrier. The particles were then tested in an Alzheimer's mouse model. The osmotin-conjugated MNPs were found to attenuate Aβ1–42-induced memory dysfunction, synaptic disorder and tau hyperphosphorylation in the hippocampus (Amin et al., 2017). These studies demonstrate that the method of administration and type/amount of magnetic field stimulation (static vs. pulsed, frequency, and strength) impact the efficiency of MNP delivery to the brain. This idea is also relevant for targeting the spinal cord, as explained below.

#### Magnetic Drug Delivery Systems Targeting the Spinal Cord

There are currently no studies that deliver drugs via MNPs to the spinal cord to promote regeneration after injury, but two studies demonstrate the potential of MNPs for this application. A preliminary study using MNPs in a T10 contusive SCI model showed that PEG-chitosan liposomes loaded with SPIONs injected via caudal vein could pass the blood-CSF barrier. This study also showed that liposome functionalization with the TAT-peptide increased the number of liposomes at the injury site. This occurred without the aid of an external magnetic field, most likely due to the compromised blood-CSF barrier that is found after injury (Wang et al., 2010). Another study looked at the effects of external magnetic field stimulation on MNPs in the spinal cord lesion. SPIONs were embedded in a 3% agarose hydrogel and implanted after complete transection of the spinal cord at the T11 vertebra. Beginning 24 h after injury, rats were exposed to a 17.96 μT, 50 Hz magnetic field for 2 h daily for 5 weeks. The lesion volume decreased by 58% in groups treated with both SPIONs and external magnetic field stimulation. Rats in this treatment group also showed improved functional recovery at each weekly timepoint; they had improved limb function, balance and stepping coordination, and sensitivity to thermal stimuli. This group also exhibited spontaneous bladder evacuation after 11 d, significantly earlier than any of the other groups. This study also showed that SPION exposure to the glioblastoma U87 cell line with a hydrogen peroxide insult improved cell viability from 44 to 75%, demonstrating the antioxidant effects of the particles (Pal et al., 2013). These two studies show the promise of MNPs for drug delivery after SCI, but more work is needed to understand the mechanisms behind magnetic field stimulation in addition to MNP delivery. MNP localization with an external magnet was demonstrated in peripheral nerves, as described in the next section.

#### Magnetic Drug Delivery Systems Targeting Peripheral Nerves

Marcus et al. tested whether MNPs could be directed and localized to peripheral nerves *in vivo*. NGF was conjugated to maghemite nanoparticles with a functionalized PEG linker. The NGF-MNPs were injected directly to the sciatic nerve and a 1-T magnetic tip placed 0.5 cm away from the injection site was positioned at a specific location along the nerve for 5 min. Dissection and staining of the nerve showed clear accumulation of MNPs at the location the magnetic tip was placed. Next, mice were given intravenous injections of the NGF-MNPs, while a 0.5-T magnet was placed near the eye for 30 min. Presence of the external magnet increased the number of MNPs in the retina by 2.5-fold, and even showed significantly higher numbers in the retina closest to the magnet compared to the opposite retina. The MNPs were also found in the liver and spleen, but not in the kidney, lungs, or brain (Marcus et al., 2018). Giannaccini et al. used polyethylenimine (PEI)-coated iron oxide nanoparticles to deliver both NGF and vascular endothelial growth factor (VEGF). Both types of functionalized particles were loaded into a 5 mm synthetic silicon conduit, which was then implanted and sutured into a 5 mm lesion created on a rat median nerve. For magnetic targeting, a 1.5 mm long piece of magnetic tape was secured at the center of the conduit, keeping the NGF- and VEGF-MNPs at the center. Only 100 ng of NGF and 5.6 ng of VEGF conjugated to MNPs showed improved nerve regeneration and motor function recovery, whereas nerves treated with the same concentration of free factors had no improvement from the control. Analysis showed that macrophages were the most prevalent cell type engulfing the MNPs in the first week, but most MNPs were found in Schwann cells by the third week after injury (Giannaccini et al., 2017). Placing the magnetic tape around the center of the conduit seemed to create a growth factor gradient that promoted cell migration to bridge the lesion and regenerate the tissue, demonstrating the advantages of precise spatial control offered by MNPs. This spatial control is also advantageous for gene therapies, allowing cells to be transfected faster and more efficiently *in vitro* and for delivery to specific tissue regions *in vivo*, as detailed below.

### Gene Delivery to the Nervous System via Magnetic Nanoparticles

Using MNPs as nucleic acid vectors to deliver nucleic acids under the influence of an external magnetic field is termed “magnetofection.” This technique has many benefits, including an improved dose-response relationship, increased delivery rates, and spatial control of delivery. Details on different formulations, techniques, and mechanisms of magnetofection have been thoroughly reviewed (Plank et al., 2011). To briefly highlight the benefits of magnetofection for neural regeneration applications, many studies originally relied on viral vectors for gene delivery to neural stem and progenitor cells; however, viral transfection is often associated with toxicity, inflammatory, and oncogenic risks, as well as non-specific cellular uptake (Puhl et al., 2019). Magnetofection has enabled successful transfection of oligodendrocyte precursor cells and neural stem/precursor cells (Jenkins et al., 2011; Pickard et al., 2011; Adams et al., 2016). As gene and cell therapies improve in efficacy and affordability, magnetofection may become paramount to the neural regeneration field.

Gene delivery to the brain is difficult because non-viral methods exhibit low transfection rates and viral vectors are usually incapable of crossing the BBB as well as risk causing an inflammatory and/or immune reaction (Niu et al., 2017). Soto-Sánchez et al. showed that neurons and other cells in the brain could be robustly transfected with a plasmid coding for yellow fluorescent protein (YFP) complexed with commercially available, positively charged iron oxide nanoparticles. A magnet was held underneath the head of the mouse for 20 min after magnetocomplexes were directly injected into the cerebral cortex. The percentage of neurons expressing YFP 3 d post-injection was 95%, and this dropped only slightly to 86% after 30 d. Animals injected with the naked plasmid showed no fluorescent signal at all. Viral vectors have a limited packaging capacity and therefore cannot carry large sequences of genetic material, whereas this study demonstrated that magnetocomplexes can deliver large sequences of genetic material to transfect neurons efficiently *in vivo* (Soto-Sánchez et al., 2015). Another study used oleic acid-coated magnetite nanoparticles conjugated with NGF via an N-isopropylacrylamide linker as a carrier for a short hairpin RNA (shRNA) against α-synuclein (α-syn) for treatment of Parkinson's disease. MNPs were injected intraperitoneally in an MPTP-induced Parkinson's mouse model and after 2 d, the number of α-syn-positive neurons decreased. MNP delivery of α-syn shRNA also improved mice behavior; distance traveled and total ambulation time and speed reached the levels of control mice (Niu et al., 2017). These studies demonstrate the potential of MNPs for improving gene delivery efficacy *in vivo*. A summary of the different *in vivo* drug and gene delivery studies is provided in Table 1.

**Table 1:** Summary of *in vivo* magnetic drug and gene delivery systems used to promote neural regeneration.

**Target tissue**	**Functional coating (dry diameter; wet diameter)**	**Drug or gene delivered**	**Magnetic stimulation**	**Application**	**References**
Brain	OQCMC and cholesterol liposomes (20 nm; 74 nm)	Paclitaxel	0.5 T magnet held over the brain for 1 h	Chemotherapy	Zhao et al., 2012
Brain	Polystyrene (10 nm; 124 nm)	Ibuprofen	0.63 T magnet secured to the animal's head	None	Kong et al., 2012
Brain	NeuroMag transfection agent (184 nm; 196 nm)	EYFP-channel rhodopsin plasmid	Magnet held beneath the brain for 20 min	Channel rhodopsin gene therapy	Soto-Sánchez et al., 2015
Brain	N-isopropylacrylamide and NGF (112 nm; 290 nm)	shRNA against α-syn	None	Parkinson's disease	Niu et al., 2017
Brain	Dextran (90 nm; 350 nm)	Osmotin	6 A, 0.5 Hz magnetic field	Alzheimer's disease	Amin et al., 2017
Spinal cord	None; embedded in agarose gel (6.5 nm; 50 nm)	None	50 Hz, 17.96 μT for 2 h daily	Complete spinal cord transection	Pal et al., 2013
Peripheral nerve	PEI (25 nm; 70 nm)	NGF and VEGF	Magnetic tape wrapped around conduit	Median nerve transection	Giannaccini et al., 2017
Peripheral nerve	PEG (18 nm; 100 nm)	NGF	1 T magnetic tip placed 0.5 cm away from injection site	Sciatic nerve injury	Marcus et al., 2018

*OQCMC, octadecyl quaternized carboxymethyl chitosan*.

### Magnetic Nanoparticle-Mediated Cell Manipulation

**Figure 2:**
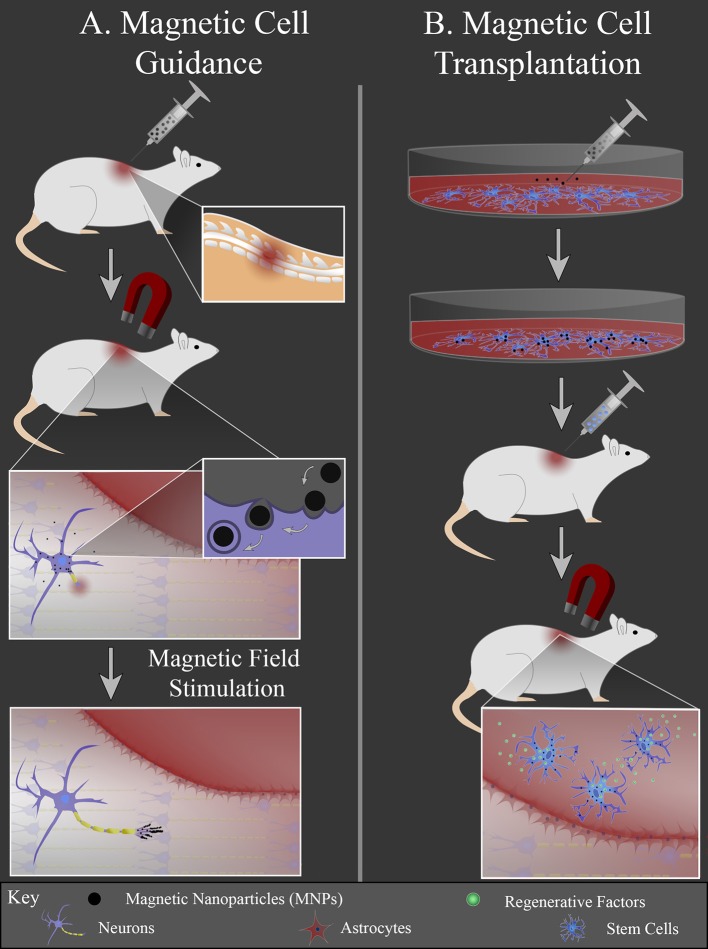
MNP-mediated cell manipulation strategies. **(A)** MNPs are injected intravenously into the animal and an external magnet is used to localize the particles at the injury site. Local cells at the injury site internalize MNPs via endocytosis. MNPs elicit local mechanical forces in response to external magnetic field stimulation, inducing axonal extension or glial cell migration into the lesion. **(B)** Cells are cultured *in vitro* and labeled with MNPs. These cells are then injected into the animal and guided to the injury site with an external magnet. Once in the lesion (red area), cells can release regenerative factors to promote regeneration.

Another area of research focuses on the interface between MNPs and cells, since cell transplantation therapies for neural injury are heavily investigated. One challenge for cell transplantation therapy is cell retention; transplanted cells must remain present at the injury site to be therapeutically effective. MNPs can be used to non-invasively guide cells to a lesion and prevent their migration away from the lesion using an external magnetic field. There are two general strategies that use MNPs to stimulate neural regeneration by manipulating cells: (1) magnetic cell guidance and (2) magnetic cell transplantation (Figure 2). For magnetic cell guidance, MNPs are injected *in vivo* and guided to a location of interest with an external magnet. The magnet is held at the location, i.e., the injury site, where the local cells either bind to or endocytose the particles. The magnetically labeled cells can then locally deliver drugs and/or be used as local mechanical stimuli. The MNPs create mechanical tension in response to a static magnetic field gradient or an alternating/pulsed magnetic field to either direct or stimulate cell growth, respectively (Figure 2A).

Magnetic cell transplantation differs in that a specific cell type is isolated and cultured *in vitro*, labeled with MNPs, and then injected *in vivo*. Following injection, cells are guided to the injury site via use of an external magnet. This approach allows the cell injection to be at an easily accessible location, since direct cell transplantation for nerve injury often requires an invasive procedure that risks further tissue damage. There can also be greater transplanted cell retention at the injury site due to the external magnetic actuation of the labeled cells (Figure 2B).

### Magnetic Nanoparticle-Induced Cell Guidance

Early studies in the field focused on how MNPs interact with neurons *in vitro*. Marcus et al. compared the interactions between PC12 cells and four different MNP types: uncoated magnetite SPIONs, uncoated maghemite SPIONs, starch-coated magnetite SPIONs, and dextran-coated magnetite SPIONs. Each particle had a hydrodynamic diameter of 100 nm. Cells were incubated with concentrations ranging from 0.01 to 0.25 mg/mL. Uncoated magnetite and starch-coated SPIONs were found bound to the outside of the cell membrane, uncoated maghemite SPIONs were internalized by cells, and the dextran-coated SPIONs did not interact with the cells. Uncoated maghemite SPIONs were detected inside cells after 1 h, with maximal loading after 24 h. Cell labeling via maghemite SPION internalization showed no effect on the PC12 cells' ability to differentiate and extend neurites. When a 0.3 T magnetic tip was placed in the center of a 35 mm culture plate for 3 d, 70% of the labeled cells were found within 3 mm of the magnetic tip, demonstrating the ability of SPION-labeled cells to migrate toward an external magnet (Marcus et al., 2016).

Riggio et al. compared the abilities of 73-nm PLL-coated magnetite SPIONs fabricated in house and commercially available fluidMAG-ARA particles produced by Chemicell to guide cell migration toward an external magnet. The ARA particles are composed of a 10 nm maghemite core coated with glucuronic acid. To better control the comparison, the group covalently bound PLL to the ARA-MNPs to create a particle with a hydrodynamic radius of 200 nm, the same hydrodynamic radius as the PLL-SPIONs. At a concentration of 10 μg/mL, both types of MNPs attached to the cell membrane and were internalized by SH-SY5Y cells (human neuroblastoma cell line) after 24 h. Both particles were also able to induce migration of the cells toward a neodymium cube magnet. The cells labeled with the homemade SPIONs were more densely populated closer to the magnet than cells labeled with the commercially available particles, potentially due to the higher magnetization saturation of the homemade particles (Riggio et al., 2012). Alon and colleagues also studied the ability of SPIONs to label PC12 cells and direct their migration. They exposed PC12 cells to 600 μg/mL gelatin-coated maghemite SPIONs for 24 h, then plated the cells on a ferromagnetic patterned substrate. Magnetic “hot spots” were generated by 350 mT bar magnets placed throughout the substrate. After 5 d of culture, the PC12 cells were preferentially distributed around the magnets, while non-labeled cells were homogeneously distributed over the substrate (Alon et al., 2015). Magnetic actuation has been utilized to achieve more complex cell patterning in other tissue engineering fields. Curved, parallel, and crossing lines of single cells as well as 3D structures have been made with this type of technique (Sensenig et al., 2012). Future studies with neural and glial magnetic cell patterning are needed to create more complex scaffolds for neural regeneration purposes.

Riggio et al. looked at how an applied magnetic field affects the orientation of PC12 cell neuronal growth processes following exposure to NGF-functionalized SPIONs. Magnetite SPIONs (25 nm diameter core) were first coated with PEI, then functionalized via NGF conjugation. The SPIONs were added to culture at 10 μg/mL and incubated for 24 h to allow interaction and induce differentiation of the PC12 cells. A cylinder magnetic applicator was then used to provide a constant magnetic field gradient of 46.5 T/m in the radial direction for 3 d. The results show that the neurites grow preferentially in the direction of the magnetic force; however, neither the SPIONs nor the magnetic field alone had an influence on the direction of neurite growth. Additionally, the neurites of cells treated with SPIONs and exposed to the magnetic field were 38% longer than those cultured without SPIONs and the magnetic field (Riggio et al., 2014). Primary leech neurons were also shown to extend neurites preferentially along a magnetic field gradient when incubated with uncoated maghemite SPIONs (Marcus et al., 2016).

Pita-Thomas and colleagues more specifically studied filopodial elongation of neurites by functionalizing MNPs with specific moieties to bind to surface molecules of retinal ganglion cells. Cholera toxin B and anti-Thy1 antibody were chemically coupled to 40-nm diameter SPIONs to target GM1-ganglioside and Thy1, respectively. They found that significantly more functionalized SPIONs were attached to retinal ganglion cell growth cones compared to non-functionalized SPIONs. Further, SPIONs remained bound to the cell membrane without being internalized. The group fabricated an electromagnetic rod with a fine tip that was placed ahead of the growth cone at a 45° angle while physically contacting the cell culture dish. The forces produced by the bound SPIONs at the filopodia tips in response to the magnetic field resulted in almost immediate elongation of the filopodia. Elongation occurred with forces as little as 3–6 pN and at 20 times the rate of normal elongation. However, elongated filopodia retracted to their original position upon removal of the magnetic force. The magnetic tip was then coated with laminin to bind the filopodia and hold it in its extended position, but this also failed to induce growth cone advance (Pita-Thomas et al., 2015). This study highlighted the complexity of filopodia function and the need for discovery of additional signaling pathways/structural components in filopodia that promote growth cone advance to design MNPs capable of inducing these pathways.

There are few studies that inject MNPs *in vivo* with the intention of engulfment by a specific cell type to elicit a response. Jain et al. fabricated negatively charged magnetic liposomes with soya lecithin, cholesterol, and phosphatidyl serine that encapsulated dextran-coated magnetite SPIONs and the anti-inflammatory drug diclofenac sodium. Additionally, liposomes were functionalized with the RGD peptide, with the goal of delivering the drug to the brain after being engulfed and carried by blood phagocytes like neutrophils and monocytes. To first test whether these cells phagocytosed the liposomes *in vivo*, liposomes were injected intravenously through the caudal vein and an 0.8 T magnetic field was applied to the selected target portion of the tail. After 2 h, blood samples were collected from the tail segment of the caudal vein where the magnetic field was applied, and the collected cell types were sorted and counted. There was a significant increase in the relative number of neutrophils and monocytes in animals treated with RGD-coated magnetic liposomes compared to non-magnetic liposomes and uncoated magnetic liposomes. To assess the liposomes' ability to deliver the anti-inflammatory drug to the brain, animals received an intra-striatal microinjection of human recombinant IL-1β to produce an inflammatory brain model. Liposomes were then administered via caudal vein at 1 mg/kg body weight and the 0.8 T magnetic field was applied near the brain of the animal for 4 h. Analysis of the relative percentage of drug found in various organs after magnetic field stimulation showed that when free drug was intravenously administered, only ~2% reached the brain. This increased to ~21% in animals treated with RGD-coated magnetic liposomes (Jain et al., 2003). Directed cell guidance *in vivo* is advantageous because it utilizes native cells, avoiding immunology issues associated with xenogenic and allogenic cell transplantation; however, this strategy has not been fully explored. Most *in vivo* work has focused on magnetic cell transplantation as a therapy for neural injury, since many believe additional cells are needed to replenish those lost at the time of injury, either by repopulating the specific cell types or by secreting factors to increase proliferation and growth.

### Magnetic Nanoparticle-Labeled Cells for Transplantation

Cell transplantation therapies have shown promise for enhancing neural repair and regeneration in animal models of nerve injury. Transplanted stem cells promote regeneration by secreting growth factors and other signaling molecules to improve cell migration and proliferation in the lesion. Stem cells are also advantageous in that they are self-renewing and can differentiate into many cell types, helping to repopulate lost cells at the injury site. Mesenchymal stem cells (MSCs) are multipotent stem cells that have been shown to provide neuroprotection and promote regeneration *in vivo*. MSCs are ideal candidates for transplantation therapies because they are relatively easy to isolate and preserve, are found abundantly in many tissues, and display low immunogenicity (Dasari et al., 2014). Bone marrow derived-MSCs (BMSCs) can be differentiated into neuronal-like cells *in vitro* and then labeled with 25 μg/mL SPIONs (Resovist—FDA-approved MRI contrast agent) without affecting their viability or differentiation state (Zhang et al., 2014). Human umbilical cord-derived MSCs have also been labeled with FDA-approved SPIONs (Feridex) at 22.4 mg Fe/mL without affecting cell viability. These cells were injected into a rat spinal cord and tracked with MRI over 14 d (Hu et al., 2009). More work is needed to determine if the MSC source produces different outcomes in nerve injury models. The culture conditions, however, of MSCs *in vitro* before transplantation were shown to impact their efficacy *in vivo*. Magnetically-labeled MSCs cultured in spheroids were compared to labeled MSCs cultured in a traditional 2D monolayer. The cells were injected into microporous PLA nerve conduits implanted to bridge a transected rat sciatic nerve and then tracked with MRI. MSCs derived from 2D culture migrated to the proximal and distal portions of the injured nerve, whereas more spheroid-derived MSCs were found at the epicenter of the conduit after 10 d. Animals treated with spheroid-derived MSCs also had the shortest nerve connection time. Interestingly, spheroid-derived MSCs could continue to be tracked with MRI up to 31 d, but 2D culture-derived cells could not (Tseng and Hsu, 2014). More work is needed to determine if the cell response to magnetic field stimulation differs in spheroid-derived or 2D culture-derived transplanted MSCs.

**Figure 3:**
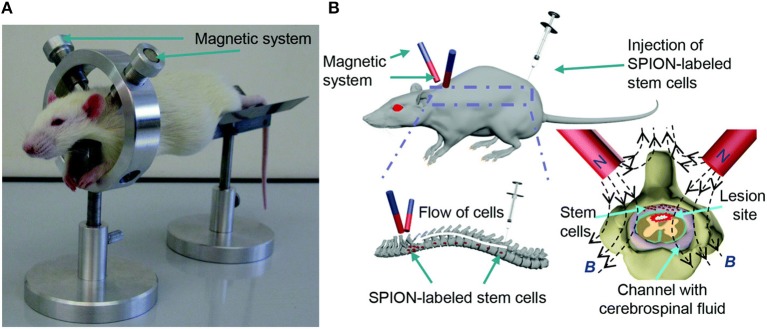
Republished with permission of RSC Pub and the Royal Society of Chemistry from Tukmachev et al. (2015) via Copyright Clearance Center. Magnetic system for MSC targeting into SCI. **(A)** *In vivo* application of the non-invasive magnetic system for MSC targeting into SCI of a rat. **(B)** Schematic representation of the magnetic targeting strategy.

MSCs have shown notable potential for improving regeneration after SCI, so the majority of magnetically-labeled cell transplantation studies for SCI utilize MSCs. One approach to magnetic cell targeting is implanting a magnet in the para-vertebral muscles at the specific site of SCI. MSCs labeled with SPIONs and administered via intrathecal injection were able to migrate through the CSF and aggregate near the magnet implant (Nishida et al., 2006; Vaněček et al., 2012). BMSCs labeled with 25 μg Fe/mL Feridex were injected into the subarachnoid space after T7 contusion SCI and were found to aggregate around a magnet implant in the paravertebral muscles after 1 d. The Basso, Beattie, and Bresnahan (BBB) locomotor test score of the magnet group significantly improved and the lesion area in the magnet group was significantly smaller than the non-magnet group 4 weeks after transplantations (Sasaki et al., 2011). Tukmachev et al. developed a two-magnet system placed on a ring-shaped holder with alike poles facing toward each other to generate a “trapping zone” at the lesion site, shown in Figure 3. PLL-coated SPIONs (140 nm hydrodynamic diameter) were used to label MSCs. One week after SCI, a half million cells were injected intrathecally 10 cm from the lesion and the magnetic system was applied for 2 h. Most of the cells were concentrated at the lesion and had even infiltrated the tissue, whereas the absence of the magnetic trapping system resulted in an even distribution of labeled cells throughout the spinal cord (Tukmachev et al., 2015). An external magnet was also able to guide MSC migration to the lesion in another study by Zhang et al. MSCs were labeled with 50 μg Fe/mL Resovist and injected into Sprague-Dawley rats via lumbar puncture 7 d after T7-8 contusion SCI. A neodymium magnet was secured externally over the SCI with medical adhesive tape. The BBB scores of the animals with the magnetic guidance were significantly higher than MSCs injected with no external magnet present after 35 d (Zhang et al., 2015). To determine the effect of pulsed magnetic field stimulation on magnetically-labeled MSCs, PEG-PE micelles containing magnetite SPIONs were incubated with MSCs at 5 μg/mL for 6 h, then injected at the caudal and cephalad portions of the injured site 7 d after T9 contusion SCI. Rats were then treated with a 50 Hz, 1 mT electromagnetic field for 5 h daily. Rats that were treated with magnetically-labeled MSCs had significantly higher BBB locomotor scores when exposed to the pulsed magnetic field after 4 weeks (Cho et al., 2013).

One clinical study has been conducted using magnetically-labeled MSCs. Autologous bone marrow CD34+ cells were extracted from patients with chronic SCI, labeled with MNPs, and delivered to the spinal cord via intrathecal transfusion. The labeled cells were detectable by MRI 35 d after injection in half of the patients in that group, whereas magnetic beads injected alone (no MSCs) were not detectable at all. The MRI showed that the labeled cells migrated to the injury site, even in the absence of an external magnetic field (Callera and de Melo, 2007). This study is the first step toward using magnetically-labeled cells to treat neural injury in humans.

Neural stem cell (NSC) transplantation has also shown promise, particularly for improving neural regeneration in the brain. Primary mouse NSCs were labeled with MNPs *in vitro* with 95% labeling efficiency and ~5.7 pg Fe/cell. Oleic acid-coated magnetite SPIONs were encapsulated in PLA with a polyvinyl alcohol coating to create MNPs with tunable amounts of magnetite. The labeled NSCs were able to differentiate into neurons, oligodendrocytes, and astrocytes. The ability of the MNPs to localize labeled NSCs was assessed in a flow system with an applied magnetic field; cells in suspension were pumped through a tube with a magnet placed at the center. Basal cell retention (cells labeled with non-magnetic particles) was ~18%, while magnetically-labeled cell retention was ~68% with 50 wt% magnetite content MNPs. Particles with 30% magnetite content only exhibited 40% cell retention at the magnet in the tube (Adams et al., 2015). Bulte et al. successfully labeled NSCs with maghemite SPIONs stabilized by carboxyl-terminated poly(amidoamine) dendrimers. Primary rat NSCs were stimulated to differentiate into oligodendrocyte progenitors and then labeled with MNPs at 25 μg Fe/mL for 24 h. These cells were then injected into both lateral ventricles of neonatal Long Evans shaker rats, animals that have a defect in the gene encoding for myelin basic protein. The labeled cells migrated into the brain parenchyma and were able to form new myelin (Bulte et al., 2001).

In a focal cerebral ischemia rat model, increased numbers of SPION-labeled NSCs injected via tail vein were found in the brain when a 0.32 T neodymium magnet was secured to the outside of the skull near the ischemic damage for 7 d. The concentration of iron was estimated to be 260 pg/cell, which did not affect viability or differentiation state of the NSCs (Song et al., 2010). Yun et al. showed that zinc-doped ferrite MNPs (ZnMNPs) activate zinc-mediated Wnt signaling in NSCs, facilitating neuronal differentiation. Their group fabricated 15-nm diameter ZnMNPs and labeled NSCs with ~4.6 pg/cell. Brain stroke was induced with a middle cerebral artery occlusion and magnetically-labeled NSCs were injected via the right internal carotid artery. A 1 T neodymium magnet was secured to the rat's head to direct the migration of the labeled-NSCs into the cortex and sub-cortex. After 5 d, cells without magnetic guidance were found in the olfactory bulb and corpus callosum, while cells with magnetic guidance were found in the cortex. Additionally, the number of viable cells delivered to the brain in the magnetically targeted group was 60-fold larger than the non-magnetically targeted group. The magnetically-labeled cells also exhibited a 2.5- and 2.2-fold increase in NGF and neurotrophin-3 (NT-3) secretion, respectively. The animals that received magnetically-labeled NSCs and external magnetic guidance had ~20% decrease in locomotor impairment score 3 weeks after transplantation (Yun et al., 2018). These studies demonstrate the ability of SPIONs to successfully label NSCs without affecting their stemness or viability, as well as the benefits of magnetically-labeled NSCs for improving neural regeneration.

Various mature cells are also studied for therapeutic transplantation in addition to stem cells; supporting glia like Schwann cells and olfactory ensheathing cells (OECs) are among the heavily investigated. One study showed that Schwann cell migration patterns could be directed with a 1.4 T neodymium magnet by labeling the cells with PLL-coated SPIONs (25 nm diameter). The investigators then tested whether this system could be used to improve Schwann cell migration into a culture of astrocytes, since Schwann cells transplanted into an injured spinal cord display poor migratory ability within the astrocyte-rich CNS. The number of magnetized Schwann cells that migrated onto an astrocyte monolayer *in vitro* increased 2.55-fold with the presence of a magnetic field compared to without field stimulation (Huang et al., 2017). In another study, primary rat Schwann cells cultured in a biodegradable chitosan-glycerophosphate polymer embedded with 28 nm diameter magnetite SPIONs were found to proliferate more when exposed to a 2-mT, 50-Hz magnetic field than without the presence of an external magnetic field. Additionally, the magnetic field stimulation increased Schwann cell expression and secretion of BDNF, GDNF, NT-3, and VEGF when cultured on the magnetic nanocomposites (Liu et al., 2014). The group continued this work by preparing a conduit filled with Schwann cells embedded in the magnetic nanocomposite scaffold. The scaffold was implanted to bridge a 15 mm long sciatic nerve gap and the animals were exposed to a 2 mT, 50 Hz magnetic field for 2 h daily. After 12 weeks, the number of myelinated axons in the nerve graft increased and better motor functional recovery was observed in animals that received magnetic stimulation with a Schwann cell-embedded conduit (MG+SCs+MF) compared to those without magnetic field stimulation (MG+SCs) or without Schwann cells (MG, MG+MF) (Liu et al., 2017). Two other studies were able to successfully label OECs with SPIONs and show their migration can be directed with an external magnet *in vitro* (Riggio et al., 2013) and tracked with MRI *in vivo* (Lee et al., 2004).

Table 2 summarizes the studies that have used SPIONs and a magnetic field to manipulate cells *in vivo*. Overall, most studies examining magnetized cells *in vivo* have used SPIONs as a contrast agent for MRI to track cell migration/position. The *in vitro* work and the few preliminary *in vivo* studies using magnetic field stimulation discussed here show the potential for SPION-assisted cell manipulation for promoting neural regeneration, in addition to improving image tracking and resolution. The diverse functions of SPIONs render them useful tools, but few have combined these functions to create a more powerful therapeutic intervention that addresses multiple aspects of regeneration. The following section will detail the few studies that have combined multiple biomaterial strategies with SPIONs and magnetic field stimulation to promote neuronal growth.

**Table 2:** SPION-assisted cell manipulation *in vivo*.

**Cell type**	**Functional coating**	**Cell guidance or transplantation**	**Magnetic stimulation**	**Application**	**References**
Macrophages	RGD-anchored liposomes carrying dextran-coated SPIONs	Magnetic cell guidance	0.8 T magnet held at the brain	Drug delivery to the brain	Jain et al., 2003
Mesenchymal stem cells	Dextran^*^	Magnetic cell transplantation	380 mT magnet implanted in paravertebral muscles	T7 contusion SCI	Sasaki et al., 2011
Mesenchymal stem cells	PLL	Magnetic cell transplantation	1.2 T magnet implanted in paravertebral muscles	T8-9 contusion SCI	Vaněček et al., 2012
Mesenchymal stem cells	PEG-PE micelles	Magnetic cell transplantation	1 mT, 50 Hz magnetic field for 5 h daily	T9 contusion SCI	Cho et al., 2013
Mesenchymal stem cells	PLL	Magnetic cell transplantation	1.2 T magnets held over lesion	T10 contusion SCI	Tukmachev et al., 2015
Mesenchymal stem cells	Carboxy-dextran^**^	Magnetic cell transplantation	Magnet externally secured at lesion	T7-8 contusion SCI	Zhang et al., 2015
Neural stem cells	Dextran^*^	Magnetic cell transplantation	320 mT magnet secured to the animal's head	Focal cerebral ischemia	Song et al., 2010
Neural stem cells	PLL ZnMNPs	Magnetic cell transplantation	1 T magnet secured to animal's head	Middle cerebral artery occlusion	Yun et al., 2018
Schwann cells	None; embedded in chitosan-glycerophosphate scaffold	Magnetic cell transplantation	2 mT, 50 Hz for 2 h daily	Sciatic nerve transection	Liu et al., 2017

*Studies that utilize an external magnetic field in addition to magnetized cells were included*.

* *Feridex is a solution of dextran-coated SPIONs and was used for this study. Feridex was approved by the FDA in 1996 as a liver imaging contrast agent, then was discontinued due to lack of sales in 2008*.

** *Resovist is a solution of carboxy-dextran-coated SPIONs and was used for this study. Resovist was approved as an imaging contrast agent by the FDA in 2001 (Cortajarena et al., 2014)*.

### Magnetic Composite Biomaterials as a Hybrid Approach for Neural Regeneration

Neural regeneration post-injury is a complex process involving many cell types and a pathophysiology timeline that requires different treatment strategies. For this reason, traumatic nerve injury in both the CNS and PNS is often very debilitating and difficult to treat. Biomaterial and cell therapy approaches are extensively studied, but the lack of significant success in animal models and clinical trials is likely because these approaches only target one or two aspects of the regeneration process, i.e., targeting one cell type or one time point in the repair and regeneration timeline. Research in this field is moving toward a hybrid approach, where multiple strategies are used together to target multiple cell types and time points.

**Figure 4:**
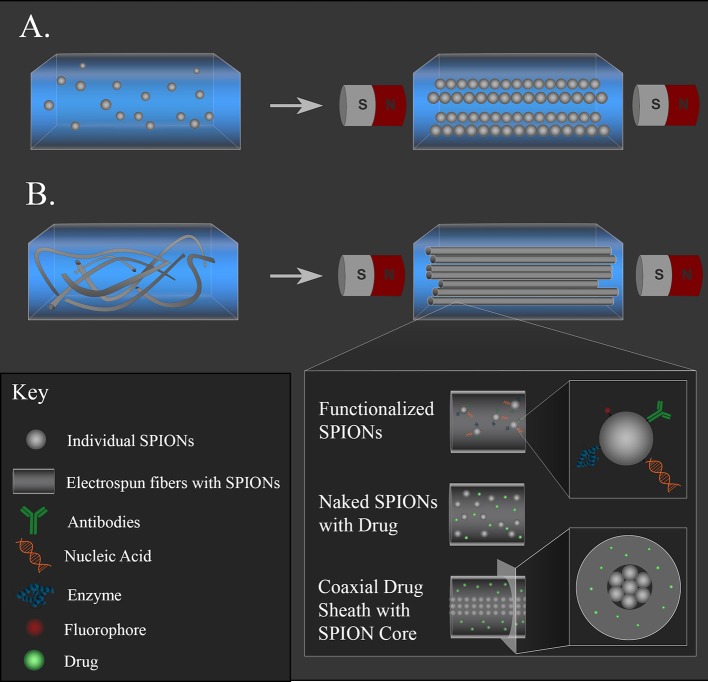
Magnetic composite materials for supporting neural regeneration and non-invasive positioning. **(A)** MNPs embedded in a hydrogel can be aligned with a magnetic field and held in place as the gel solidifies. **(B)** MNP-loaded fibers can be aligned with a magnetic field and held in place as a gel solidifies. The MNPs can be functionalized with drugs, antibodies, nucleic acids, or fluorophores in either system. Naked MNPs can be loaded alongside drugs in the hydrogel or in the polymer fiber solution before electrospinning. Coaxial fibers with MNP-loaded core and drug-containing sheath can also be fabricated to offer magnetic alignment with tunable fiber nanotopography.

Composite constructs often consist of hydrogels with either magnetic particles embedded within the hydrogel network (Figure 4A) or fibers that were electrospun with MNPs (Figure 4B). These composite materials are promising approaches for neural regeneration, as both types allow for non-invasive alignment via an external magnetic field of either particles or fibers, which provide topographical cues to direct neurite outgrowth. MNPs are easily functionalized with drugs, antibodies, or nucleic acids, or these therapeutics can be incorporated into polymer coatings on the surface of the MNPs to extend release. Functionalized MNPs can then be incorporated into a hydrogel or fibers to combine topographical cues with therapeutic molecule delivery. Different therapeutic molecules can also be incorporated into the different materials (gel, particle coating, polymer fibers) so that they are released at rates that align with certain physiological time points. For example, an anti-inflammatory can be mixed in the hydrogel solution so that it is acutely released during the initial stage of inflammation after injury, while a neurotrophic drug can be incorporated into a polymer fiber to be released over weeks while damaged axons regrow. The following studies demonstrate the diversity of composites that can be fabricated.

Antman-Passig and Shefi embedded SPIONs in a collagen liquid suspension that was then allowed to solidify in a static magnetic field. They controlled the aggregation of the particles in the gel by tuning the gelation rate and the strength of the external magnetic field, forming strings of magnetic particles that follow the magnetic field lines (depicted in Figure 4A). Antman-Passig and Shefi were also able to alter the magnetic particles and gel formation dynamics to achieve alignment of the collagen fibers as they formed. To achieve this, uncoated magnetite and maghemite particles with 50 or 100 nm hydrodynamic diameters were compared as well as dextran, starch, or citric acid salt-coated MNPs. They found that 100-nm magnetite particles demonstrated better collagen fiber alignment than all the other particles tested, so this formulation was studied with a culture of primary leech neurons dispersed throughout the gel. A low magnetic field (255 G) was applied for 40 min, and cells were cultured in the gel for 7 d. Cells within the oriented gels led to significantly elongated neuronal shape with an aspect ratio close to 3, twice the ratio of the control. The majority of the cells aligned with the topography that was directed by the magnetic field; 60% of the cells produced neurites aligned within 15° of the magnetic field lines (Antman-Passig and Shefi, 2016).

Rose et al. took a slightly different approach to producing magnetically-responsive topography within a hydrogel. Magnetic rod-shaped PEG microgels were fabricated with a mold-based soft lithography approach, then embedded in a macroscopic hydrogel matrix that could be crosslinked to fix the oriented microgels in place. A concentration of 400 μg/mL SPIONs, corresponding to 0.0046 vol%, were homogeneously distributed in microgels with dimensions 50 × 5 × 5 μm^3^. In a 100 mT magnetic field, these microgels aligned in ~36 s. Increasing the SPION content or the magnetic field strength resulted in shorter orientation times, but microgels began to aggregate, so the 400 μg/mL SPION concentration and 100 mT field were used in cell experiments. The microgels were dispersed within a human fibrin hydrogel in various concentrations with chick DRG for 5 d to determine the cell response to the microgel topography cues. A mean inter-microgel distance of 34 μm in the fibrin gel (1 v/v%) was enough to orient neurite outgrowth. Increasing the microgel content did not significantly enhance neurite guidance, and concentrations lower than 1% microgels in the fibrin hydrogel did not result in neurite alignment. Overall, this design allows precise control over microgel dimensions, shape, stiffness, porosity, and water and SPION content so that the overall construct can be tailored to different macro- and micro-environments (Rose et al., 2017).

Aligned electrospun fibers have been shown to significantly increase neurite outgrowth compared to gels, acting as a substrate for cell adhesion. In an early study, Kriha et al. added superparamagnetic cobalt nanoparticles into electrospun poly(methyl methacrylate-vinyl acetate) copolymer fibers. They demonstrated that 50–100 μm fiber fragments could be manipulated with a 1.4 T ferromagnet to position the fibers between two hippocampal neurons, prompting connection (Kriha et al., 2007). As electrospun fibers require an invasive procedure to implant at the injury site to maintain fiber alignment, two groups aimed to increase the translational efficacy of fibers by developing magnetic fibers that can be injected and then aligned with a magnetic field. Omidinia-Anarkoli et al. created an injectable hydrogel with short magnetic fibers that orient in a low strength (<300 mT) magnetic field. Aligned PLGA fibers were electrospun with 10 wt/wt% SPIONs and cut into 100-μm long segments. Fiber diameter was 700 nm, while particle diameter was 5 nm. The fiber segments were then mixed in a fibrinogen solution and subjected to a 100 mT magnetic field, while thrombin was added to form a fibrin gel, immobilizing the aligned fibers within it. Chick DRG grown in the gels for 7 d produced neurites that aligned with magnetically oriented fibers, whereas neurites extended radially in gels without fibers. Neurite extension increased by 55% for aligned short fibers compared to gels without any fibers. This demonstrates the ability of short fibers to be injected and magnetically oriented to enhance neurite outgrowth through a gel (Omidinia-Anarkoli et al., 2017).

Johnson et al. aimed to improve upon this approach by using millimeter-scale fibers to provide a more continuous contact guidance compared to micro-scale fibers dispersed through a gel. PLLA fibers were electrospun with various percentages (0–8 w/w%) of oleic acid-coated SPIONs (12 nm diameter). The fiber mats were then cut into 5–6 mm segments and rolled into conduits. These conduits were loaded into a 22-G needle and injected into a well *in vitro*; magnetic conduits fabricated with 6% SPION electrospun fibers aligned with a 1.42 T magnetic field in 0.87 s after injection into either collagen (2 mg/mL) or fibrinogen (10 mg/mL) solutions. Additionally, primary rat neurons from dissociated DRG cultured on SPION-containing fibers yielded a significant increase in the mean neurite outgrowth compared to the control PLLA fibers. A 3D neurite outgrowth model was tested by injecting a single conduit of 6% SPION fibers into the collagen or fibrinogen solutions containing a suspended DRG. The conduit was oriented using a magnet so that the end of one side of the conduit was in contact with the DRG body and the long axis of the conduit extended away. Neurites that contacted the fibers oriented in the same direction as the fibers, and the average neurite length on the fibers increased 1.4 times (collagen) or 3 times (fibrin) compared to neurites that extended into the gel alone (Johnson et al., 2018). The ability to remotely control the positioning of a larger construct within a viscous solution is a promising first step for implementation of magnetic materials *in vivo*. Though preliminary, these studies attempt to improve upon biomaterial strategies for promoting neural regeneration by incorporating magnetic elements. Magnetic coaxial fibers (depicted in Figure 4) have also been fabricated (Song et al., 2006; Sung et al., 2012; Lee et al., 2015), but there are currently no published studies that have used magnetic coaxial fibers for neural regeneration applications. Future studies are needed to develop these types of composite materials and determine their efficacy. The next section will consider the general challenges moving forward with magnetic composite materials.

## Challenges Associated With Magnetic Materials for Clinical Applications

Despite the recent advances in magnetic composite materials described here, there are a few major challenges that must be addressed before these types of therapies make it to the clinic. First, the optimal magnetic field stimulation paired with the type, size, shape, and functional coating of SPIONs for promoting neural regeneration is currently unknown. Different magnetic carriers travel differently through blood and tissues and the same type of magnetic carrier experiences different motion resistance based on the type of bodily fluid or tissue that it is moving through. For example, 100 nm chitosan-coated SPIONs penetrated deeper into liver tissue than kidney or brain tissue in the presence of a 0.4 T magnetic field *in vitro*. The study also showed that 500-nm chitosan-coated SPIONs penetrated deeper in the liver, penetrated to the same depth in the kidney, and penetrated less deep in the brain, as compared to their 1-μm sized counterparts (Kulkarni et al., 2015). Therefore, optimization of different particle parameters (size, coating, etc.) for specific applications is critical. Additionally, different aspects of magnetic field stimulation should be considered, including the strength of the field, whether it is a static, pulsed, or alternating field, and the duration of stimulation. If the field stimulation is dynamic, the stimulation frequency can have varying effects as well. For example, higher frequencies (30–300 kHz) are used for cancer hyperthermia, where stimulation of MNPs generates localized heating to kill tumor cells (Stigliano et al., 2013). In contrast, lower frequencies (<100 Hz) have been shown to activate mechanosenstive ion channels and promote cell growth (Hughes et al., 2005). However, less is known about the upper limit of safe frequency stimulation since heat generation of moving particles in different tissues is not well-characterized (Kalambur et al., 2005). In terms of strength, MRI systems most commonly found in hospitals today are either 1.5 or 3 T. However, 7 T systems are becoming more common, providing higher-resolution images without causing tissue damage (Nowogrodzki, 2018). It is currently unknown how MRI machines can be utilized to move SPIONs through the body or at what concentrations and pulse frequencies does SPION movement become detrimental to cell viability. Table 3 summarizes some of the important parameters to consider when designing magnetic composite materials; however, this list is not exhaustive. There are many possible combinations of magnetic field and particle parameters, so mathematical modeling and simulations may be necessary to provide guidance for more efficient optimization.

**Table 3:** Parameters to consider when designing magnetic composite materials.

**Magnetic particles**	**Magnetic field stimulation**	**Application**
Material	Strength	Type of animal model
Size	Type (static, pulsed, alternating)	Therapeutic molecules
Surface coating	Frequency (if dynamic)	Release rate of therapeutics
	Duration	Method of administration

Another major challenge is effectively penetrating deep tissues with magnetic composite materials, since an external magnet's strength decreases quickly with distance. This can be achieved by either improving external magnets to provide stronger and deeper magnetic gradients or by optimizing the magnetic materials to react more strongly in a magnetic gradient (Shapiro et al., 2015). The composite approach may be beneficial to increasing the magnetic susceptibility of the materials; for example, incorporating higher concentrations of MNPs in electrospun fibers linearly increased the fibers' saturation magnetization (Johnson et al., 2018). Additionally, MRI systems can be improved to provide higher resolution tracking of MNPs, which will help to determine how these particles move *in vivo*.

There will be further challenges related to the translation of these composite materials into animal models and clinical trials. Contusive injuries are the most clinically relevant type of nervous system injury; however, injecting composite materials into a swollen and inflamed tissue and remotely controlling their positioning without further damage to the tissue will not be a trivial task. To show potential efficacy, a first step could be to demonstrate that magnetic composite materials can be remotely manipulated *in vivo* within a transection injury. However, for these materials to be clinically relevant for human use, efficacy in crush injuries will also need to be established.

## Conclusion

Research in neural regeneration is moving toward hybrid approaches, as current pharmacological and surgical approaches lack efficacy. The complex pathophysiology of nervous system injury requires therapies that target multiple aspects of the repair and regeneration process for various durations. Magnetic composite materials offer a wide range of design parameters that can be tailored to achieve this. MNPs can be coated with drugs, growth factors, or nucleic acids, or these therapeutics can be incorporated into polymer coatings on the surface of the MNPs to extend release. Functionalized MNPs can then be embedded within a hydrogel or electrospun fibers or internalized by cells used for transplantation therapy or by native cells *in vivo*. These types of composite materials offer many possible combinations of physical and chemical cues that can be tailored to multiple different cell types and/or timescales of drug release to target specific events in the pathophysiology timeline. Overall, there is a need for collaboration between engineers, chemists, biologists, mathematicians, and clinicians to overcome the challenges mentioned above and generate composite magnetic materials that can be translated for human use.

## Author Contributions

JF planned, wrote, and reviewed the manuscript. BB created figures and aided in writing. RG reviewed and provided comments for the manuscript.

### Conflict of Interest Statement

The authors declare that the research was conducted in the absence of any commercial or financial relationships that could be construed as a potential conflict of interest.

## References

[B1] Adams C. Israel L. L. Ostrovsky S. Taylor A. Poptani H. Lellouche J.-P.. (2016). Development of multifunctional magnetic nanoparticles for genetic engineering and tracking of neural stem cells. Adv. Healthc. Mater. 5, 841–849. 10.1002/adhm.201500885 26867130

[B2] Adams C. F. Rai A. Sneddon G. Yiu H. H. Polyak B. Chari D. M. (2015). Increasing magnetite contents of polymeric magnetic particles dramatically improves labeling of neural stem cell transplant populations. Nanomed. Nanotechnol. Biol. Med. 11, 19–29. 10.1016/j.nano.2014.07.001 25038496

[B3] Aguilera G. Berry C. C. West R. M. Gonzalez-Monterrubio E. Angulo-Molina A. Arias-Carrión Ó. (2019). Carboxymethyl cellulose coated magnetic nanoparticles transport across a human lung microvascular endothelial cell model of the blood–brain barrier. Nanoscale Adv. 1, 671–685. 10.1039/C8NA00010G PMC9473188 36132237

[B4] Albayar A. A. Roche A. Swiatkowski P. Antar S. Ouda N. Emara E.. (2019). Biomarkers in spinal cord injury: prognostic insights and future potentials. Front. Neurol. 10:27. 10.3389/fneur.2019.00027 30761068 PMC6361789

[B5] Alizadeh A. Dyck S. M. Karimi-Abdolrezaee S. (2019). Traumatic spinal cord injury: an overview of pathophysiology, models and acute injury mechanisms. Front. Neurol. 10:282. 10.3389/fneur.2019.00282 30967837 PMC6439316

[B6] Almad A. Sahinkaya F. R. McTigue D. M. (2011). Oligodendrocyte fate after spinal cord injury. Neurotherapeutics 8, 262–273. 10.1007/s13311-011-0033-5 21404073 PMC3101831

[B7] Alon N. Havdala T. Skaat H. Baranes K. Marcus M. Levy I.. (2015). Magnetic micro-device for manipulating PC12 cell migration and organization. Lab. Chip 15, 2030–2036. 10.1039/C5LC00035A 25792133

[B8] Alvites R. Caseiro A. R. Pedrosa S. S. Branquinho M. V. Ronchi G. Geuna S. (2018). Peripheral nerve injury and axonotmesis: state of the art and recent advances. Cogent Med. 5:1466404 10.1080/2331205X.2018.1466404

[B9] Amin F. U. Hoshiar A. K. Do T. D. Noh Y. Shah S. A. Khan M. S.. (2017). Osmotin-loaded magnetic nanoparticles with electromagnetic guidance for the treatment of Alzheimer's disease. Nanoscale 9, 10619–10632. 10.1039/C7NR00772H 28534925

[B10] Antman-Passig M. Shefi O. (2016). Remote magnetic orientation of 3D collagen hydrogels for directed neuronal regeneration. Nano Lett. 16, 2567–2573. 10.1021/acs.nanolett.6b00131 26943183

[B11] Arslantunali D. Dursun T. Yucel D. Hasirci N. Hasirci V. (2014). Peripheral nerve conduits: technology update. Med. Devices 7, 405–424. 10.2147/MDER.S59124 25489251 PMC4257109

[B12] Barres B. A. Jacobson M. D. Schmid R. Sendtner M. Raff M. C. (1993). Does oligodendrocyte survival depend on axons? Curr. Biol. 3, 489–497. 10.1016/0960-9822(93)90039-Q 15335686

[B13] Bellver-Landete V. Bretheau F. Mailhot B. Vallières N. Lessard M. Janelle M. E.. (2019). Microglia are an essential component of the neuroprotective scar that forms after spinal cord injury. Nat. Commun. 10:518. 10.1038/s41467-019-08446-0 30705270 PMC6355913

[B14] Bollaerts I. Van houcke J. Andries L. De Groef L. Moons L. (2017). Neuroinflammation as fuel for axonal regeneration in the injured vertebrate central nervous system. Mediat. Inflamm. 2017:9478542. 10.1155/2017/9478542 28203046 PMC5288536

[B15] Bulte J. W. Douglas T. Witwer B. Zhang S. C. Strable E. Lewis B. K.. (2001). Magnetodendrimers allow endosomal magnetic labeling and *in vivo* tracking of stem cells. Nat. Biotechnol. 19, 1141–1147. 10.1038/nbt1201-1141 11731783

[B16] Burda J. E. Bernstein A. M. Sofroniew M. V. (2016). Astrocyte roles in traumatic brain injury. Exp. Neurol. 275, 305–315. 10.1016/j.expneurol.2015.03.020 25828533 PMC4586307

[B17] Callera F. de Melo C. M. (2007). Magnetic resonance tracking of magnetically labeled autologous bone marrow CD34+ cells transplanted into the spinal cord via lumbar puncture technique in patients with chronic spinal cord injury: CD34+ cells' migration into the injured site. Stem Cells Dev. 16, 461–466. 10.1089/scd.2007.0083 17610376

[B18] Cangellaris O. V. Gillette M. U. (2018). Biomaterials for enhancing neuronal repair. Front. Mater. 5:21 10.3389/fmats.2018.00021

[B19] Canning D. R. Höke A. Malemud C. J. Silver J. (1996). A potent inhibitor of neurite outgrowth that predominates in the extracellular matrix of reactive astrocytes. Int. J. Dev. Neurosci. 14, 153–175. 10.1016/0736-5748(96)00004-4 8842795

[B20] Casha S. Yu W. R. Fehlings M. G. (2001). Oligodendroglial apoptosis occurs along degenerating axons and is associated with FAS and p75 expression following spinal cord injury in the rat. Neuroscience 103, 203–218. 10.1016/S0306-4522(00)00538-8 11311801

[B21] Cho H. Choi Y. K. Lee D. H. Park H. J. Seo Y. K. Jung H.. (2013). Effects of magnetic nanoparticle-incorporated human bone marrow–derived mesenchymal stem cells exposed to pulsed electromagnetic fields on injured rat spinal cord. Biotechnol. Appl. Biochem. 60, 596–602. 10.1002/bab.1109 24033637

[B22] Ciofani G. Raffa V. Menciassi A. Cuschieri A. Micera S. (2009). Magnetic alginate microspheres: system for the position controlled delivery of nerve growth factor. Biomed. Microdevices 11, 517–527. 10.1007/s10544-008-9258-4 19067172

[B23] Cortajarena A. L. Ortega D. Ocampo S. M. Gonzalez-García A. Couleaud P. Miranda R.. (2014). Engineering iron oxide nanoparticles for clinical settings. Nanobiomedicine 1:2. 10.5772/58841 30023013 PMC6029241

[B24] D'Agata F. Ruffinatti F. A. Boschi S. Stura I. Rainero I. Abollino O.. (2017). Magnetic nanoparticles in the central nervous system: targeting principles, applications and safety issues. Molecules 23:E9. 10.3390/molecules23010009 29267188 PMC5943969

[B25] Dasari V. R. Veeravalli K. K. Dinh D. H. (2014). Mesenchymal stem cells in the treatment of spinal cord injuries: a review. World J. Stem Cells 6, 120–133. 10.4252/wjsc.v6.i2.120 24772239 PMC3999770

[B26] Dilnawaz F. Sahoo S. K. (2015). Therapeutic approaches of magnetic nanoparticles for the central nervous system. Drug Discov. Today 20, 1256–1264. 10.1016/j.drudis.2015.06.008 26103617

[B27] Estelrich J. Escribano E. Queralt J. Busquets M. A. (2015). Iron oxide nanoparticles for magnetically-guided and magnetically-responsive drug delivery. Int. J. Mol. Sci. 16, 8070–8101. 10.3390/ijms16048070 25867479 PMC4425068

[B28] Fournier A. E. GrandPre T. Strittmatter S. M. (2001). Identification of a receptor mediating Nogo-66 inhibition of axonal regeneration. Nature 409, 341–346. 10.1038/35053072 11201742

[B29] Frati A. Cerretani D. Fiaschi A. I. Frati P. Gatto V. La Russa R.. (2017). Diffuse axonal injury and oxidative stress: a comprehensive review. Int. J. Mol. Sci. 18:E2600. 10.3390/ijms18122600 29207487 PMC5751203

[B30] Giannaccini M. Calatayud M. P. Poggetti A. Corbianco S. Novelli M. Paoli M.. (2017). Magnetic nanoparticles for efficient delivery of growth factors: stimulation of peripheral nerve regeneration. Adv. Healthc. Mater. 6:1601429. 10.1002/adhm.201601429 28156059

[B31] Gilbert R. J. McKeon R. J. Darr A. Calabro A. Hascall V. C. Bellamkonda R. V. (2005). CS-4,6 is differentially upregulated in glial scar and is a potent inhibitor of neurite extension. Mol. Cell. Neurosci. 29, 545–558. 10.1016/j.mcn.2005.04.006 15936953

[B32] Gupta A. K. Gupta M. (2005). Synthesis and surface engineering of iron oxide nanoparticles for biomedical applications. Biomaterials 26, 3995–4021. 10.1016/j.biomaterials.2004.10.012 15626447

[B33] Hola K. Markova Z. Zoppellaro G. Tucek J. Zboril R. (2015). Tailored functionalization of iron oxide nanoparticles for MRI, drug delivery, magnetic separation and immobilization of biosubstances. Biotechnol. Adv. 33, 1162–1176. 10.1016/j.biotechadv.2015.02.003 25689073

[B34] Hu S. L. Zhang J. Q. Hu X. Hu R. Luo H. S. Li F.. (2009). *In vitro* labeling of human umbilical cord mesenchymal stem cells with superparamagnetic iron oxide nanoparticles. J. Cell. Biochem. 108, 529–535. 10.1002/jcb.22283 19623584

[B35] Huang L. Nakamura Y. Lo E. H. Hayakawa K. (2019). Astrocyte signaling in the neurovascular unit after central nervous system injury. Int. J. Mol. Sci. 20:282. 10.3390/ijms20020282 30642007 PMC6358919

[B36] Huang L. Xia B. Liu Z. Cao Q. Huang J. Luo Z. (2017). Superparamagnetic iron oxide nanoparticle-mediated forces enhance the migration of schwann cells across the astrocyte-schwann cell boundary *in vitro*. Front. Cell. Neurosci. 11:83. 10.3389/fncel.2017.00083 28400720 PMC5368970

[B37] Huebner E. A. Strittmatter S. M. (2009). Axon regeneration in the peripheral and central nervous systems. Results Probl. Cell Differ. 48, 339–351. 10.1007/400_2009_19 19582408 PMC2846285

[B38] Hughes S. El Haj A. J. Dobson J. (2005). Magnetic micro- and nanoparticle mediated activation of mechanosensitive ion channels. Med. Eng. Phys. 27, 754–762. 10.1016/j.medengphy.2005.04.006 15985383

[B39] Jain S. Mishra V. Singh P. Dubey P. K. Saraf D. K. Vyas S. P. (2003). RGD-anchored magnetic liposomes for monocytes/neutrophils-mediated brain targeting. Int. J. Pharm. 261, 43–55. 10.1016/S0378-5173(03)00269-2 12878394

[B40] Jeffery N. D. McBain S. C. Dobson J. Chari D. M. (2009). Uptake of systemically administered magnetic nanoparticles (MNPs) in areas of experimental spinal cord injury (SCI). J. Tissue Eng. Regen. Med. 3, 153–157. 10.1002/term.139 19051217

[B41] Jenkins S. I. Pickard M. R. Granger N. Chari D. M. (2011). Magnetic nanoparticle-mediated gene transfer to oligodendrocyte precursor cell transplant populations is enhanced by magnetofection strategies. ACS Nano 5, 6527–6538. 10.1021/nn2018717 21721568

[B42] Johnson C. D. L. Ganguly D. Zuidema J. M. Cardinal T. J. Ziemba A. M. Kearns K. R.. (2018). Injectable, magnetically orienting electrospun fiber conduits for neuron guidance. ACS Appl. Mater. Interfaces 11, 356–372. 10.1021/acsami.8b18344 30516370 PMC6520652

[B43] Kalambur V. S. Han B. Hammer B. E. Shield T. W. Bischof J. C. (2005). *In vitro* characterization of movement, heating and visualization of magnetic nanoparticles for biomedical applications. Nanotechnology 16, 1221–1233. 10.1088/0957-4484/16/8/041

[B44] Karve I. P. Taylor J. M. Crack P. J. (2016). The contribution of astrocytes and microglia to traumatic brain injury. Br. J. Pharmacol. 173, 692–702. 10.1111/bph.13125 25752446 PMC4742296

[B45] Kim J. A. Lee N. Kim B. H. Rhee W. J. Yoon S. Hyeon T.. (2011). Enhancement of neurite outgrowth in PC12 cells by iron oxide nanoparticles. Biomaterials 32, 2871–2877. 10.1016/j.biomaterials.2011.01.019 21288566

[B46] Kong S. D. Lee J. Ramachandran S. Eliceiri B. P. Shubayev V. I. Lal R.. (2012). Magnetic targeting of nanoparticles across the intact blood–brain barrier. J. Control. Release 164, 49–57. 10.1016/j.jconrel.2012.09.021 23063548 PMC4440873

[B47] Kong X. Gao J. (2017). Macrophage polarization: a key event in the secondary phase of acute spinal cord injury. J. Cell. Mol. Med. 21, 941–954. 10.1111/jcmm.13034 27957787 PMC5387136

[B48] Kriha O. Becker M. Lehmann M. Kriha D. Krieglstein J. Yosef M. (2007). Connection of hippocampal neurons by magnetically controlled movement of short electrospun polymer fibers—a route to magnetic micromanipulators. Adv. Mater. 19, 2483–2485. 10.1002/adma.200601937

[B49] Kudr J. Haddad Y. Richtera L. Heger Z. Cernak M. Adam V.. (2017). Magnetic nanoparticles: from design and synthesis to real world applications. Nanomaterials 7:243. 10.3390/nano7090243 28850089 PMC5618354

[B50] Kulkarni S. Ramaswamy B. Horton E. Gangapuram S. Nacev A. Depireux D.. (2015). Quantifying the motion of magnetic particles in excised tissue: effect of particle properties and applied magnetic field. J. Magn. Magn. Mater. 393, 243–252. 10.1016/j.jmmm.2015.05.069 26120240 PMC4477713

[B51] Lee H. J. Lee S. J. Uthaman S. Thomas R. G. Hyun H. Jeong Y. Y.. (2015). Biomedical applications of magnetically functionalized organic/inorganic hybrid nanofibers. Int. J. Mol. Sci. 16, 13661–13677. 10.3390/ijms160613661 26084046 PMC4490516

[B52] Lee I.-H. Bulte J. W. M. Schweinhardt P. Douglas T. Trifunovski A. Hofstetter C.. (2004). *In vivo* magnetic resonance tracking of olfactory ensheathing glia grafted into the rat spinal cord. Exp. Neurol. 187, 509–516. 10.1016/j.expneurol.2004.02.007 15144877

[B53] Liddelow S. A. Barres B. A. (2017). Reactive astrocytes: production, function, and therapeutic potential. Immunity 46, 957–967. 10.1016/j.immuni.2017.06.006 28636962

[B54] Liu X. Z. Xu X. M. Hu R. Du C. Zhang S. X. McDonald J. W.. (1997). Neuronal and glial apoptosis after traumatic spinal cord injury. J. Neurosci. 17, 5395–5406. 10.1523/JNEUROSCI.17-14-05395.1997 9204923 PMC6793816

[B55] Liu Z. Huang L. Liu L. Luo B. Liang M. Sun Z.. (2014). Activation of Schwann cells *in vitro* by magnetic nanocomposites via applied magnetic field. Int. J. Nanomed. 10, 43–61. 10.2147/IJN.S74332 25565803 PMC4275057

[B56] Liu Z. Zhu S. Liu L. Ge J. Huang L. Sun Z.. (2017). A magnetically responsive nanocomposite scaffold combined with Schwann cells promotes sciatic nerve regeneration upon exposure to magnetic field. Int. J. Nanomed. 12, 7815–7832. 10.2147/IJN.S144715 29123395 PMC5661463

[B57] Lutz A. B. Chung W.-S. Sloan S. A. Carson G. A. Zhou L. Lovelett E. (2017). Schwann cells use TAM receptor-mediated phagocytosis in addition to autophagy to clear myelin in a mouse model of nerve injury. *Proc. Natl. Acad. Sci*. U. S. A. 114, E8072–E8080. 10.1073/pnas.1710566114 PMC5617301 28874532

[B58] Marcus M. Karni M. Baranes K. Levy I. Alon N. Margel S.. (2016). Iron oxide nanoparticles for neuronal cell applications: uptake study and magnetic manipulations. J. Nanobiotechnol. 14:37. 10.1186/s12951-016-0190-0 27179923 PMC4867999

[B59] Marcus M. Skaat H. Alon N. Margel S. Shefi O. (2014). NGF-conjugated iron oxide nanoparticles promote differentiation and outgrowth of PC12 cells. Nanoscale 7, 1058–1066. 10.1039/C4NR05193A 25473934

[B60] Marcus M. Smith A. Maswadeh A. Shemesh Z. Zak I. Motiei M.. (2018). Magnetic targeting of growth factors using iron oxide nanoparticles. Nanomaterials 8:E707. 10.3390/nano8090707 30201889 PMC6163445

[B61] McBain S. C. Yiu H. H. Dobson J. (2008). Magnetic nanoparticles for gene and drug delivery. Int. J. Nanomed. 3, 169–180. 10.2147/IJN.S1608 18686777 PMC2527670

[B62] McKeon R. J. Schreiber R. C. Rudge J. S. Silver J. (1991). Reduction of neurite outgrowth in a model of glial scarring following CNS injury is correlated with the expression of inhibitory molecules on reactive astrocytes. J. Neurosci. 11, 3398–3411. 10.1523/JNEUROSCI.11-11-03398.1991 1719160 PMC6575543

[B63] McKerracher L. David S. Jackson D. L. Kottis V. Dunn R. J. Braun P. E. (1994). Identification of myelin-associated glycoprotein as a major myelin-derived inhibitor of neurite growth. Neuron 13, 805–811. 10.1016/0896-6273(94)90247-X 7524558

[B64] Menorca R. M. G. Fussell T. S. Elfar J. C. (2013). Peripheral nerve trauma: mechanisms of injury and recovery. Hand Clin. 29, 317–330. 10.1016/j.hcl.2013.04.002 23895713 PMC4408553

[B65] Mukhopadhyay G. Doherty P. Walsh F. S. Crocker P. R. Filbin M. T. (1994). A novel role for myelin-associated glycoprotein as an inhibitor of axonal regeneration. Neuron 13, 757–767. 10.1016/0896-6273(94)90042-6 7522484

[B66] Nectow A. R. Marra K. G. Kaplan D. L. (2012). Biomaterials for the development of peripheral nerve guidance conduits. Tissue Eng. Part B Rev. 18, 40–50. 10.1089/ten.teb.2011.0240 21812591 PMC3262974

[B67] Nishida K. Tanaka N. Nakanishi K. Kamei N. Hamasaki T. Yanada S.. (2006). Magnetic targeting of bone marrow stromal cells into spinal cord: through cerebrospinal fluid. Neuroreport 17, 1269–1272. 10.1097/01.wnr.0000227993.07799.a2 16951567

[B68] Niu S. Zhang L.-K. Zhang L. Zhuang S. Zhan X. Chen W.-Y.. (2017). Inhibition by multifunctional magnetic nanoparticles loaded with alpha-synuclein RNAi plasmid in a Parkinson's disease model. Theranostics 7, 344–356. 10.7150/thno.16562 28042339 PMC5197069

[B69] Nowogrodzki A. (2018). The world's strongest MRI machines are pushing human imaging to new limits. Nature 563, 24–26. 10.1038/d41586-018-07182-7 30382222

[B70] NSCISC (2019). NSCISC Facts and Figures. Available online at: https://www.nscisc.uab.edu

[B71] Omidinia-Anarkoli A. Boesveld S. Tuvshindorj U. Rose J. C. Haraszti T. De Laporte L. (2017). An injectable hybrid hydrogel with oriented short fibers induces unidirectional growth of functional nerve cells. Small 13:1702207. 10.1002/smll.201702207 28783255

[B72] Oyinbo C. A. (2011). Secondary injury mechanisms in traumatic spinal cord injury: a nugget of this multiply cascade. Acta Neurobiol. Exp. 71, 281–299. 21731081 10.55782/ane-2011-1848

[B73] Pal A. Singh A. Nag T. C. Chattopadhyay P. Mathur R. Jain S. (2013). Iron oxide nanoparticles and magnetic field exposure promote functional recovery by attenuating free radical-induced damage in rats with spinal cord transection. Int. J. Nanomed. 8, 2259–2272. 10.2147/IJN.S44238 23818782 PMC3693820

[B74] Pickard M. R. Barraud P. Chari D. M. (2011). The transfection of multipotent neural precursor/stem cell transplant populations with magnetic nanoparticles. Biomaterials 32, 2274–2284. 10.1016/j.biomaterials.2010.12.007 21193228

[B75] Pilakka-Kanthikeel S. Atluri V. S. R. Sagar V. Saxena S. K. Nair M. (2013). Targeted Brain Derived Neurotropic Factors (BDNF) delivery across the blood-brain barrier for neuro-protection using magnetic nano carriers: an *in-vitro* study. PLoS ONE 8:62241. 10.1371/journal.pone.0062241 23653680 PMC3639992

[B76] Pita-Thomas W. Steketee M. B. Moysidis S. N. Thakor K. Hampton B. Goldberg J. L. (2015). Promoting filopodial elongation in neurons by membrane-bound magnetic nanoparticles. Nanomed. Nanotechnol. Biol. Med. 11, 559–567. 10.1016/j.nano.2014.11.011 25596077 PMC4691347

[B77] Plank C. Zelphati O. Mykhaylyk O. (2011). Magnetically enhanced nucleic acid delivery. Ten years of magnetofection-progress and prospects. Adv. Drug Deliv. Rev. 63, 1300–1331. 10.1016/j.addr.2011.08.002 21893135 PMC7103316

[B78] Popovich P. G. Guan Z. Wei P. Huitinga I. van Rooijen N. Stokes B. T. (1999). Depletion of hematogenous macrophages promotes partial hindlimb recovery and neuroanatomical repair after experimental spinal cord injury. Exp. Neurol. 158, 351–365. 10.1006/exnr.1999.7118 10415142

[B79] Puhl D. L. D'Amato A. R. Gilbert R. J. (2019). Challenges of gene delivery to the central nervous system and the growing use of biomaterial vectors. Brain Res. Bull. 150, 216–230. 10.1016/j.brainresbull.2019.05.024 31173859 PMC8284997

[B80] Quraishe S. Forbes L. H. Andrews M. R. (2018). The extracellular environment of the cns: influence on plasticity, sprouting, and axonal regeneration after spinal cord injury. Neural Plast. 2018:2952386. 10.1155/2018/2952386 29849554 PMC5932463

[B81] Reis C. Gospodarev V. Reis H. Wilkinson M. Gaio J. Araujo C.. (2017). Traumatic brain injury and stem cell: pathophysiology and update on recent treatment modalities. Stem Cells Int. 2017:6392592. 10.1155/2017/6392592 28852409 PMC5568618

[B82] Riggio C. Calatayud M. P. Giannaccini M. Sanz B. Torres T. E. Fernández-Pacheco R.. (2014). The orientation of the neuronal growth process can be directed via magnetic nanoparticles under an applied magnetic field. Nanomed. Nanotechnol. Biol. Med. 10, 1549–1558. 10.1016/j.nano.2013.12.008 24407149

[B83] Riggio C. Calatayud M. P. Hoskins C. Pinkernelle J. Sanz B. Torres T. E.. (2012). Poly-l-lysine-coated magnetic nanoparticles as intracellular actuators for neural guidance. Int. J. Nanomed. 7, 3155–3166. 10.2147/IJN.S28460 22811603 PMC3394465

[B84] Riggio C. Nocentini S. Catalayud M. P. Goya G. F. Cuschieri A. Raffa V.. (2013). Generation of magnetized olfactory ensheathing cells for regenerative studies in the central and peripheral nervous tissue. Int. J. Mol. Sci. 14, 10852–10868. 10.3390/ijms140610852 23708092 PMC3709706

[B85] Robinson L. R. (2000). Traumatic injury to peripheral nerves. Muscle Nerve 23, 863–873. 10.1002/(SICI)1097-4598(200006)23:6<863::AID-MUS4>3.0.CO;2-0 10842261

[B86] Rose J. C. Cámara-Torres M. Rahimi K. Köhler J. Möller M. De Laporte L. (2017). Nerve cells decide to orient inside an injectable hydrogel with minimal structural guidance. Nano Lett. 17, 3782–3791. 10.1021/acs.nanolett.7b01123 28326790 PMC5537692

[B87] Rotshenker S. (2011). Wallerian degeneration: the innate-immune response to traumatic nerve injury. J. Neuroinflamm. 8:109. 10.1186/1742-2094-8-109 21878125 PMC3179447

[B88] Sasaki H. Tanaka N. Nakanishi K. Nishida K. Hamasaki T. Yamada K.. (2011). Therapeutic effects with magnetic targeting of bone marrow stromal cells in a rat spinal cord injury model. Spine 36, 933–938. 10.1097/BRS.0b013e3181eb9fb0 21217457

[B89] Sensenig R. Sapir Y. MacDonald C. Cohen S. Polyak B. (2012). Magnetic nanoparticle-based approaches to locally target therapy and enhance tissue regeneration *in vivo*. Nanomed. 7, 1425–1442. 10.2217/nnm.12.109 22994959 PMC3543693

[B90] Shapiro B. Kulkarni S. Nacev A. Muro S. Stepanov P. Y. Weinberg I. N. (2015). Open challenges in magnetic drug targeting. Wiley Interdiscip. Rev. Nanomed. Nanobiotechnol. 7, 446–457. 10.1002/wnan.1311 25377422 PMC4397114

[B91] Song M. Kim Y.-J. Kim Y. Roh J. Kim S. U. Yoon B.-W. (2010). Using a neodymium magnet to target delivery of ferumoxide-labeled human neural stem cells in a rat model of focal cerebral ischemia. Hum. Gene Ther. 21, 603–610. 10.1089/hum.2009.144 20059319

[B92] Song T. Zhang Y. Z. Zhou T. J. (2006). Fabrication of magnetic composite nanofibers of poly(ε-caprolactone) with FePt nanoparticles by coaxial electrospinning. J. Magn. Magn. Mater. 303, e286–e289. 10.1016/j.jmmm.2006.01.247

[B93] Soto-Sánchez C. Martínez-Navarrete G. Humphreys L. Puras G. Zarate J. Pedraz J. L.. (2015). Enduring high-efficiency *in vivo* transfection of neurons with non-viral magnetoparticles in the rat visual cortex for optogenetic applications. Nanomed. Nanotechnol. Biol. Med. 11, 835–843. 10.1016/j.nano.2015.01.012 25680542

[B94] Stigliano R. V. Shubitidze F. Kekalo K. Baker I. Giustini A. J. Hoopes P. J. (2013). Understanding mNP Hyperthermia for cancer treatment at the cellular scale. Proc. SPIE– Int. Soc. Opt. Eng. 8584:85840E. 10.1117/12.2007518 25249755 PMC4169898

[B95] Sung Y. K. Ahn B. W. Kang T. J. (2012). Magnetic nanofibers with core (Fe3O4 nanoparticle suspension)/sheath (poly ethylene terephthalate) structure fabricated by coaxial electrospinning. J. Magn. Magn. Mater. 324, 916–922. 10.1016/j.jmmm.2011.03.004

[B96] Thomsen L. B. Thomsen M. S. Moos T. (2015). Targeted drug delivery to the brain using magnetic nanoparticles. Ther. Deliv. 6, 1145–1155. 10.4155/tde.15.56 26446407

[B97] Tseng T.-C. Hsu S. (2014). Substrate-mediated nanoparticle/gene delivery to MSC spheroids and their applications in peripheral nerve regeneration. Biomaterials 35, 2630–2641. 10.1016/j.biomaterials.2013.12.021 24388817

[B98] Tukmachev D. Lunov O. Zablotskii V. Dejneka A. Babic M. Syková E.. (2015). An effective strategy of magnetic stem cell delivery for spinal cord injury therapy. Nanoscale 7, 3954–3958. 10.1039/C4NR05791K 25652717

[B99] Vaněček V. Zablotskii V. Forostyak S. Rurička J. Herynek V. Babič M.. (2012). Highly efficient magnetic targeting of mesenchymal stem cells in spinal cord injury. Int. J. Nanomed. 7, 3719–3730. 10.2147/IJN.S32824 22888231 PMC3414205

[B100] Vargas M. E. Barres B. A. (2007). Why is wallerian degeneration in the CNS so slow? Annu. Rev. Neurosci. 30, 153–179. 10.1146/annurev.neuro.30.051606.094354 17506644

[B101] Wang H. Zhang S. Liao Z. Wang C. Liu Y. Feng S.. (2010). PEGlated magnetic polymeric liposome anchored with TAT for delivery of drugs across the blood-spinal cord barrier. Biomaterials 31, 6589–6596. 10.1016/j.biomaterials.2010.04.057 20553983

[B102] Wen X. Wang K. Zhao Z. Zhang Y. Sun T. Zhang F.. (2014). Brain-targeted delivery of trans-activating transcriptor-conjugated magnetic PLGA/Lipid nanoparticles. PLoS ONE 9:6652. 10.1371/journal.pone.0106652 25187980 PMC4154764

[B103] Yun S. Shin T. H. Lee J. H. Cho M. H. Kim I. S. Kim J.. (2018). Design of magnetically labeled cells (Mag-Cells) for *in vivo* control of stem cell migration and differentiation. Nano Lett. 18, 838–845. 10.1021/acs.nanolett.7b04089 29393650

[B104] Zhang R. Li J. Li J. Xie J. (2014). Efficient *in vitro* labeling rabbit bone marrow-derived mesenchymal stem cells with spio and differentiating into neural-like cells. Mol. Cells 37, 650–655. 10.14348/molcells.2014.0010 25234466 PMC4179133

[B105] Zhang R. Xu C. Liu Y. Li J. Xie J. (2015). Visual bone marrow mesenchymal stem cell transplantation in the repair of spinal cord injury. Neural Regen. Res. 10, 404–411. 10.4103/1673-5374.153688 25878588 PMC4396102

[B106] Zhao M. Chang J. Fu X. Liang C. Liang S. Yan R.. (2012). Nano-sized cationic polymeric magnetic liposomes significantly improves drug delivery to the brain in rats. J. Drug Target. 20, 416–421. 10.3109/1061186X.2011.651726 22519867

[B107] Ziemba A. M. Gilbert R. J. (2017). Biomaterials for local, controlled drug delivery to the injured spinal cord. Front. Pharmacol. 8:245. 10.3389/fphar.2017.00245 28539887 PMC5423911

[B108] Zigmond R. E. Echevarria F. D. (2019). Macrophage biology in the peripheral nervous system after injury. Prog. Neurobiol. 173, 102–121. 10.1016/j.pneurobio.2018.12.001 30579784 PMC6340791

[B109] Ziv-Polat O. Shahar A. Levy I. Skaat H. Neuman S. Fregnan F.. (2014). The role of neurotrophic factors conjugated to iron oxide nanoparticles in peripheral nerve regeneration: *in vitro* studies. BioMed Res. Int. 2014:267808. 10.1155/2014/267808 25133160 PMC4123480

[B110] Zuidema J. M. Gilbert R. J. Gottipati M. K. (2018). Biomaterial approaches to modulate reactive astroglial response. Cells Tissues Org. 205, 372–395. 10.1159/000494667 30517922 PMC6397084

[B111] Zuidema J. M. Provenza C. Caliendo T. Dutz S. Gilbert R. J. (2015). Magnetic NGF-releasing PLLA/iron oxide nanoparticles direct extending neurites and preferentially guide neurites along aligned electrospun microfibers. ACS Chem. Neurosci. 6, 1781–1788. 10.1021/acschemneuro.5b00189 26322376

